# The Role of Intrinsically Unstructured Proteins in Neurodegenerative Diseases

**DOI:** 10.1371/journal.pone.0005566

**Published:** 2009-05-15

**Authors:** Swasti Raychaudhuri, Sucharita Dey, Nitai P. Bhattacharyya, Debashis Mukhopadhyay

**Affiliations:** 1 Structural Genomics Section, Saha Institute of Nuclear Physics, Kolkata, India; 2 Crystallography and Molecular Biology Division, Saha Institute of Nuclear Physics, Kolkata, India; Swiss Federal Institute of Technology Lausanne, Switzerland

## Abstract

The number and importance of intrinsically disordered proteins (IUP), known to be involved in various human disorders, are growing rapidly. To test for the generalized implications of intrinsic disorders in proteins involved in Neurodegenerative diseases, disorder prediction tools have been applied to three datasets comprising of proteins involved in Huntington Disease (HD), Parkinson's disease (PD), Alzheimer's disease (AD). Results show, in general, proteins in disease datasets possess significantly enhanced intrinsic unstructuredness. Most of these disordered proteins in the disease datasets are found to be involved in neuronal activities, signal transduction, apoptosis, intracellular traffic, cell differentiation etc. Also these proteins are found to have more number of interactors and hence as the proportion of disorderedness (i.e., the length of the unfolded stretch) increased, the size of the interaction network simultaneously increased. All these observations reflect that, “Moonlighting” i.e. the contextual acquisition of different structural conformations (transient), eventually may allow these disordered proteins to act as network “hubs” and thus they may have crucial influences in the pathogenecity of neurodegenerative diseases.

## Introduction

Of late there has been a considerable shift in the protein sequence-structure-function paradigm. With the ever emerging population of Disordered, Natively Unfolded or Intrinsically Unstructured Proteins (IUPs), it is now generally understood that the structure-function correlation is a contextual phenomenon for a protein molecule. For a metabolic enzyme with an “ordered” structure, the particular conformation required for “induced fit” may have a very high negative conformational free energy chosen by evolutionary selection pressure over time and thus the issue of “context” may appear to be irrelevant in these cases. Although exceptions to these classical views had been reported earlier where a well characterized enzyme was found to have altogether different function in a different context, and conform to a somewhat polymorphic model [1]–[3].

IUPs do not form a fixed three dimensional structure under physiological conditions either in their entireties or they may contain Intrinsically Disordered Regions (IDRs). Their structures resemble the denatured states of ordered proteins, best described as an ensemble of rapidly interconverting alternative structures, which nevertheless, are their native, functional states [4]. They take up different structures upon binding to different targets, and thereby exhibit functional flexibility through the formation of fuzzy complexes [2]. The extent of structural variations, upon functional binding of a ligand, ranges from slight conformational adjustments seen in allosterism to a drastic conformational switch or loss of structure [1]. Interestingly it is also known that intrinsic disorder is more prevalent (35–51%) in eukaryotic organisms whereas only 7–33% and 9–37% of bacteria and archaea proteins, respectively, contain long unstructured regions as calculated by disorder prediction tool PONDR VL-XT [5]. Paradoxically this observation conforms to the fact that the number of components in the genome and the proteome for an organism are uncorrelated. Considering the enormous complexity of functions that a eukaryotic proteome needs to handle starting with the information from a single gene sequence, IUPs provide another level of variation in its portfolio in addition to other known events like alternative splicing or post-translational modifications. Understandably, this is the case for higher organisms having limited genome size.

The conformational promiscuity or “pliability” of the IUPs makes them capable of “multitasking” or “moonlighting” [3]. Although these proteins lack regular structures, the IUPs carry out important biological functions including regulation of cell division, chaperone activity, signaling and transcriptional and translational control [6]. In an intricate protein-protein interaction network, they, therefore, play the role of “hubs” or “nodes” and provide “robustness”. From a systems biology point of view, alterations (e.g., mutations) in the genes coding for the “hub” protein would not be advantageous as they might lead to partial or complete collapse of the network. This network “failure” might, in turn, lead to several functional abnormalities promulgating disease pathogenesis. Evaluating the involvement and influence of IUPs in monogenic as well as multifactorial complex disorders of late onset type may give us important clues about the disease mechanisms.

Recently, it has been found that disorder is very common in complex human diseases. Iakoucheva et al. predicted, using the neural network predictor PONDR VL-XT, that 79% of the cancer associated proteins contain regions of disorder of ≥30 residues. In contrast, only 13% of proteins from a set of proteins with well-defined ordered structures contain such long regions of predicted disorder [6]. In the same study 66% of cellular signaling associated proteins were found to be enriched in disorder. In another study using the same tool, Cheng et al. have shown that 57% of the cardiovascular disease associated proteins contain 30 or more consecutive residues, predicted to be disordered [7]. They also used PONDR VL-XT as the disorder prediction tool. Thus disorder was found to be significantly higher among these disease-associated proteins than the total pool of eukaryotic proteins in SwissProt database, 47% of which contained disordered regions by the same definition. In another study, Cheng et al., using the same algorithm, estimated that among human disease-associated proteins, including autoimmune diseases, cancer, cardiovascular diseases, diabetes, and neurodegenerative diseases, approximately 69% were expected to contain disorder regions of ≥30 consecutive amino acid residues using PONDR VL-XT algorithm [8]. Among those disease associated proteins, 21% were identified to have roles in cell signaling pathways and were found to contain long disordered regions, compared to rest 8% of the cell signaling proteins that were predicted to be predominately ordered. For the entire set of disease-associated proteins with long disordered regions, 48% were predicted to be not participating in signaling [8].

The group of neurodegenerative disorders currently comprise of about 32 known types of different diseases. Interestingly, in many of the neurodegenerative diseases, the common feature is misfolding and aggregation of proteins, the major contributory factor of neurotoxicity [9]. They exert toxicity by disrupting intracellular transport, overwhelming protein degradation pathways, and/or disturbing vital cell functions [10]. Some of the key proteins that cause neurodegenerative diseases like APP, SNCA or Htt contain IDRs or they themselves are IUPs [11]–[17]. Recently we have provided evidence that HYPK, an interacting partner of Htt, whose mutation causes HD, is an IUP [11]. It would be interesting therefore to evaluate the potential role of IUPs in disease processes [18]. In this article, we have dealt with three of them; e.g. Huntington's disease (HD), Alzheimer's disease (AD) and Parkinson's disease (PD). They are the commonest among human neurodegenerative diseases, significantly affecting a large population [19]. HD is an autosomal dominant disease caused by a trinucleotide (CAG) repeat expansion beyond 36 in the Huntingtin (*htt*) gene that produces an altered form of the Htt protein. The elongated poly Q sequence thus produced is believed to initiate protein misfolding resulting in nuclear aggregation in the cells of striatum and cortex [20], [21]. Alzheimer's disease is characterized by the presence of two lesions: the plaque, an extracellular lesion made up largely of the β-amyloid (Aβ) peptide, and the tangle, an intracellular lesion made up largely of the cytoskeletal protein tau. The pathological hallmark of Parkinson's disease is the deposition of Lewy bodies, cytoplasmic inclusions composed largely of α-synuclein, within the dopaminergic neurons [19]. Using bioinformatics tools here we have characterized the IUPs involved in these diseases and analyzed their functional significance.

## Results

### Unstructured Proteins are Prevalent in Neurodegenerative Diseases

Following the protocols described in the methods section, we developed six independent datasets. After stringent filtering of the retrieved data from literature and interaction databases, three disease datasets, named “HD dataset”, “PD dataset”, and “AD dataset”, were generated. Three control datasets were also constructed. “Control dataset 1” comprised of 17159 hits from SwissProt (release 55.0), “control dataset 2” comprised of 264 human enzymes which have known PDB structures and “control dataset 3” consisted of 117 human proteins implicated in breast cancer which were also derived from SwissProt.

Using the described selection criteria, the disorder indices of the proteins in all six independently constructed datasets were calculated. It was immediately apparent that unstructuredness was significantly (at 95% level of significance) prevalent among proteins of “AD dataset” and “HD dataset” with respect to the “control dataset 1”. For the “PD dataset”, however, the prevalence was not significant (the data was found to be significant at the 90% level) with respect to the “control dataset 1”. The “control dataset 2” was constructed to ensure that “unstructuredness” may not be necessary for all the genetic diseases, for example in metabolic disorders, where the involved proteins are predominantly structured enzymes. When compared to “control dataset 2”, unstructuredness was found to be significantly prevalent in all the disease datasets. We constructed the “control dataset 3” to check whether the involvement of IUPs was specific for neurodegenerative diseases or it generally related to disease. Intriguingly, unstructuredness was not significantly enriched (at 95% level of significance) in the “control dataset 3”, which comprised of proteins implicated in breast cancer, compared to “control dataset 1”; whereas in comparison to “control dataset 2” it was significant. The summary of these results, along with the Z scores calculated is shown in **Table 1**.

**Table 1:** Prevalence of Unstructured proteins in HD, PD and AD.

Type	Size of dataset	Percentage unstructuredness	Z values* [C1]	Z values* [C2]
HD	147	81.6	1.80	6.8
PD	65	75.4	NS#	3.3
AD	140	80.7	1.94	5.3
Control dataset 1 [C1]	17159	73.4	–	–
Control dataset 2 [C2]	264	47.7	6.39	–
Control dataset 3 [C3]	117	76.9	NS#	4.6

* Z tests were done at 95% level of significance [52].

# NS: Not Significant.

**Figure 1:**
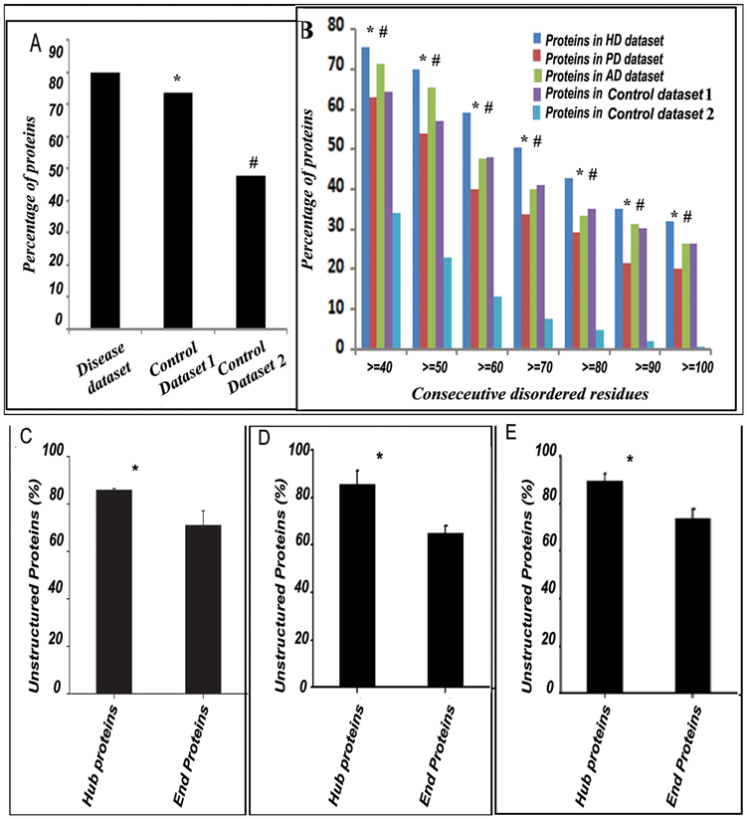
Intrinsically unstructured proteins are prevalent in neurodegenerative disease dataset. A: Considering all the 352 members of the disease datasets, about 80% of the proteins were found to be unstructured compared to 73.4% and 47.7% in “control datasets 1 and 2” respectively. Level of significance was calculated by Z-test and the Z-values indicate that unstructuredness is highly prevalent in the disease dataset compared to “control datasets 1 and 2” (denoted by * and #). B: The percentage of proteins in HD, PD and AD datasets with ≥40 to ≥100 consecutive residues unstructured compared to “control datasets 1 and 2”. Levels of significance was calculated by Z-test throughout indicating significant prevalence of proteins having ≥40 to ≥100 consecutive residues unstructured in the HD datasets compared to “control dataset 1” and denoted by $ in each cases. In PD and AD dataset no such significant prevalence was observed. However, compared to “control dataset 2” proteins having ≥40 to ≥100 consecutive residues unstructured are significantly enriched in HD, PD and AD datasets and denoted by #. C, D & E: Unstructuredness is significantly prevalent in the “hub” proteins involved in neurodegenerative diseases. All the proteins in HD, PD and AD datasets were analyzed for the presence of “hub” and “end” proteins and the percentage of IUPs among the “hub” and “end” proteins in the disease datasets were calculated and plotted. Irrespective of the definition of “hub” (protein with ≥10 interactors (C), protein with ≥5 interactors (D) or protein with ≥20 interactors (E), IUPs were significantly prevalent among “hub” proteins. Levels of significance were calculated by Student's t-test and P values were 0.014 (C), 0.011 (D) and 0.012 (E) respectively, indicated by * in each panel.

When we considered all the 352 proteins that have experimentally validated implications in HD, PD or AD (i.e., a non-redundant combination of “HD dataset”, “PD dataset” and “AD dataset” – designated as “disease dataset”), we found ∼80% of them were unstructured compared to 73.4% and 47.7% in “control dataset 1 and 2” respectively **(** **Figure 1A** **)**. Z values calculated for the 352 proteins compared to “control dataset 1 and 2” were 3.27 and 6.5 respectively signifying prevalence (at 99% level of significance) of unstructured proteins in disease datasets. It was then prudent to ask whether the proteins designated as “disordered” in the disease datasets contained lengthier unstructured regions compared to the disordered proteins in “control dataset 1 and 2”. We plotted percentage of proteins containing disordered regions against the number of consecutive amino acids residues predicted to be disordered and found in comparison to “control dataset 1”, a significant prevalence of lengthier unstructured regions in proteins from “HD dataset” only but compared to “control dataset 2”, all the disease datasets were enriched in lengthier unstructured proteins. **(** **Figure 1B** **)**.

### Unstructuredness is Prevalent in Hub Proteins in the HD, PD and AD datasets

There is an intuition that with increasing disorderedness, number of interactors of a protein would also increase [22], [23]. In order to see whether the number of interactors of a protein increased with unstructuredness, we initially counted the numbers of “hub” and “end” proteins among the proteins which have experimentally validated influence in disease processes and present in HD, PD and AD datasets. Almost 49% (72 out of 147), 51% (33 out of 65) and 53% (74 out of 140) proteins of HD, PD and AD datasets respectively were found to have more than 10 interactors (hub proteins). Among these hub proteins 92, 85 and 88% of the proteins in HD, PD and AD datasets, respectively, were found to be unstructured. In contrast, 74, 65 and 76% of the end proteins in HD, PD and AD datasets respectively were unstructured. When we considered all the hub and end proteins in these three disease datasets together, unstructured proteins were found to be significantly (P value = 0.0133) prevalent among the hub proteins in the disease datasets (88.3%) compared to the end proteins (71.6%) **(** **Figure 1C** **)**.

However, to ensure that all possible biasness was eliminated, we used a validation protocol where the same calculations were done with varying cutoff values to define “hub” and “end”. Interestingly, with these independent analyses also a similar trend was observed. Here, rather than defining a protein with more than 10 interactors as a “hub”, those with more than 5 or 20 interactors in the disease datasets were defined as “hubs” respectively (similarly those with less than 5 or 20 interactors were termed “ends” respectively), in two consecutive cases, and the “hub proteins” from all these three datasets were considered together to estimate the number of unstructured proteins among them. Almost 86% of the “hub proteins” were found to be unstructured when proteins with more than 5 interactors were defined as “hubs” **(** **Figure 1D** **)**. In contrast 65% of the “end proteins” were found to be unstructured. Likewise 89.6% of the “hub proteins” were found to be unstructured when proteins with more than 20 interactors were called “hubs”. In contrast 73.8% of the “end proteins” were found to be unstructured **(** **Figure 1E** **)**.

### Functions of the IUPs in Neurodegenerative Diseases (HD, PD and AD)

We carried out functional annotations of the proteins in the disease datasets using the “Biological Process” annotation tool available in “PANTHER” database. Primarily we looked for the processes which were significantly enriched in disease proteins compared to total human proteome dataset. Subsequently we investigated whether those processes were enriched with unstructured proteins or not. Interestingly, processes like neuronal activities, signal transduction, cell cycle, intracellular traffic, apoptosis, protein targeting and metabolism etc., appeared to be the major processes involved in disease pathogenesis. Proteins participating in these processes were significantly enriched in disease datasets as well as they were populated with unstructured proteins.

**Figure 2:**
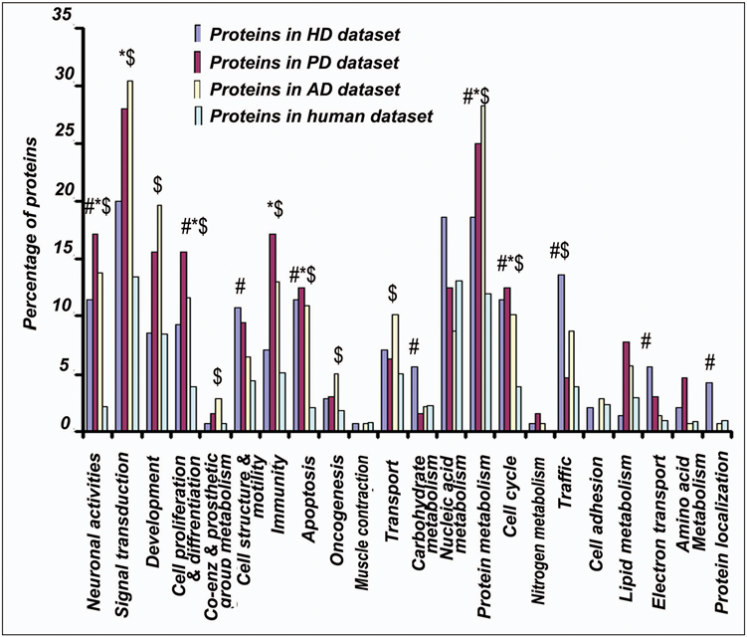
Functional annotations of the proteins in disease datasets. All the protein IDs for HD, PD and AD datasets were submitted separately in batch to annotate the biological process by the “PANTHER biological process” annotation tool and the results were tabulated and analyzed. Biological processes that were significantly enriched with proteins in HD, PD and AD protein datasets compared to total human proteome dataset are indicated in figure by # (for HD), * (for PD) and $ (for AD). Level of significance in each case was calculated by Chi-square test. Chi-square test was performed and p values were calculated with the aid of “GraphPad QuickCalcs” (http://www.graphpad.com/quickcalcs/chisquared1.cfm).

**Figure 3:**
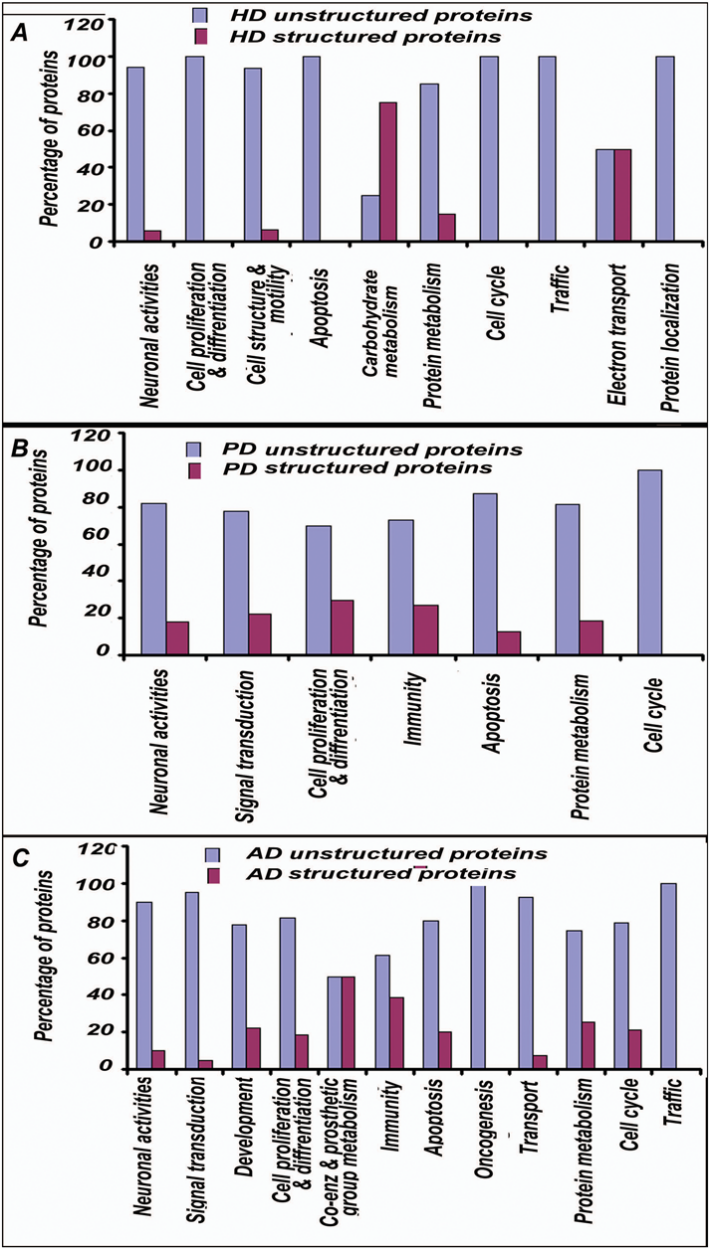
Prevalence of unstructuredness in biological processes enriched with proteins from HD, PD and AD datasets. A: Except for carbohydrate metabolism and electron transport, proteins involved in all other important biological processes, as indicated, are significantly enriched in unstructured proteins in HD. Levels of significance were calculated by Z-test indicating confidence level of 95% in each case. B: Proteins involved in all the important processes in PD as shown in figure are significantly enriched in unstructured proteins. Levels of significance were calculated by Z-test indicating confidence level of 95% in each case. C: Except processes like coenzyme and prosthetic group metabolism and immunity and defense, proteins involved in all other important processes in AD, as mentioned, are significantly enriched in unstructured proteins.

The proteins in “HD dataset” were found to have significantly enriched participation in “biological processes” like neuronal activities, cell proliferation and differentiation, cell structure and motility, apoptosis and its regulation, carbohydrate metabolism, protein metabolism, cell cycle, intracellular protein traffic, electron transport and protein targeting and localization etc. **(** **Figure 2** **)**. When the contribution of unstructuredness among these proteins was investigated, interestingly, it was found that except for carbohydrate metabolism and electron transport, proteins involved in all other important processes in the HD were significantly enriched in unstructured proteins **(** **Figure 3A** **)**.

Likewise proteins in “PD dataset” were found to have significantly enriched participation in neuronal activities, signal transduction, cell proliferation and differentiation, immunity and defense, apoptosis and its regulation, protein metabolism, cell cycle **(** **Figure 2** **)** and the proteins involved in these processes were largely unstructured **(** **Figure 3B** **)**.

Analysis of the proteins in “AD dataset” revealed significantly enriched participation in neuronal activities, signal transduction, developmental processes, cell proliferation and differentiation, coenzyme and prosthetic group metabolism, immunity and defense, apoptosis and its regulation, oncogenesis, transport, protein metabolism and modification, cell cycle and intracellular protein traffic etc **(** **Figure 2** **)**. Barring processes like coenzyme and prosthetic group metabolism and immunity and defense, proteins involved in all other important processes in the AD were significantly unstructured **(** **Figure 3C** **)**.

## Discussion

Computational estimates suggest that eukaryotic proteomes have a significantly higher occurrence of disordered proteins relative to prokaryotic proteomes [4]. The prevalence of intrinsically unstructured proteins in eukaryotes is likely to be due to more complex signaling and regulatory pathways that heavily rely on disordered proteins [24]. While much has been studied about the general mechanisms of protein-protein interactions, the specific structural features that account for differences in protein interactivity has recently been ascribed to “fuzzy” complex formation and are largely unknown [2]. In case of disordered proteins this interaction network is much more complex and vast compared to that of the ordered/globular proteins [24]. These proteins do not have a fixed structure and hence they are flexible with a tendency to interact with many other proteins.

It has been established by other groups that partially or fully disorderd proteins are prevalent in complex disorders like neurodegenerative diseases [25], cancer, cardiovascular disease or diabetes [8] and led to the “disorder in disorder (D2)” concept [26]. Here we have investigated the content of unstructuredness in HD, PD and AD datasets, the commonest of neurodegenerative diseases, which shows significantly high prevalence of unstructured proteins in most of these diseases and the extent of unstructuredness is comparable to the previous reports. Proteins in Huntington's disease (HD dataset) were found to be most unstructured. HD being a monogenic disorder, this is somewhat expected as all the pathologically important proteins should interact with the single hub. In case of AD or PD, where several genes are implicated, multiple hubs should be there and we considered only one such hub. It has earlier been reported that a correlation exists between unstructuredness and the complexity of an organism [4], [27]. It is speculated that for a particular protein, the more it is unstructured, the more is its possibility to interact with diverse partners, and hence the interaction network of the protein becomes large and complex [28]. Analysis of unstructuredness among HD, PD and AD dataset reveals that the hub proteins in these datasets (those interacting with 10, 5 or 20 partners) are more unstructured than the end proteins **(** **Figures 1C, 1D and 1E** **)**. It has been previously reported that hubs should have more unstructured residues [22]. Our findings from a different perspective conform to that. It also subscribes to the evolutionary model of network organization by hub proteins [23], possibly through “fuzzy” complex formation [2]. It can be suggested that in a disease protein network, loss of interactions around the hub over time could ankylose the network leading to its failure. However, this could just be an indirect consequence of the prevalence of unstructured proteins in the network and hence it would be premature to guess the impacts of “hubs” on such networks.

Interestingly, the types of “biological processes”, where the components of the individual datasets are found to participate significantly, are very much characteristic of the functional/phenotypic imbalances recorded for the concerned diseases. We found that components participating in these biological processes are also enriched with unstructuredness. Consequently, it could be summarized that not only these neurodegenerative disease datasets are enriched in unstructured proteins but also most of these unstructured proteins play pivotal role in the disease pathogenesis by participating in vital biological processes.

Conventional wisdom suggests that genes known to cause disease should predominantly encode “hubs” [29]–[31]. Expanding their analysis to include human orthologs of mouse genes involved in pre- or post-natal lethality, Goh K.–I. et al. [32], however, observed an “unexpected peripherality” of the disease causing genes in the sense that essential genes were found to be clustered into “hubs” and were not involved in diseases. In a later study Feldman I. et al. [33] pointed out that the correlation between the parameters “essentiality” and “connectivity” should not be deterministic. Using a bigger size of data (Goh K. -I. et al. used a limited number of data from OMIM) Feldman I et al. did notice functional clustering of disease genes despite the limited knowledge of the human interactome. In our study, excepting the single disease causing genes for each disorder, we did not categorize other components in terms of their ability to cause disease. Rather we focused on constructing the network in terms of protein structural disorder and the “essentiality” parameter has not been considered. How exactly the clustering, that we observed, would be correlated to “essentiality” is not a simple question to address and would demand a separate analysis. But in the IUP scenario, where the proteins are multitasking and the functions are context based, it would be interesting to apply Goh et al.'s approach and to see how the boundary between the “disease” and “essential” genes are drawn.

Interestingly, even if the disorder in the overall “disease dataset” was significantly high **(** **Figure 1A** **)** in comparison to “control dataset 1”, the “PD dataset” failed to show this behavior. This is probably due to insufficient experimental data available for PD or the interactome of α-synuclein could be highly underrepresented in the databases. In recent times a lot of efforts are being given in understanding the biology of PD [34 and references therein] and it is realized that the disease may be an outcome of the dynamic interplay between α-synuclein and its close homologue β-synuclein, both unstructured proteins. The discrepancy that we noticed in the PD dataset may also be a result of a different interaction pattern in this disease. Similar trend was observed in breast cancer proteins when “control dataset 3” was compared to “control dataset 1” although it is reported that unstructuredness is prevalent in cancer [6]. It is to be noted further that we considered the human proteome alone as control for our analysis, whereas a selection of the whole eukaryotic proteome would have less disorder, as it was the case (∼35–51%) in the analysis by Dunker A.K. et al. [4] using PONDR tools, since the disorder contribution from lower eukaryotes was much less there. To justify, we used a “biased” “control dataset 2” (comprising of metabolic enzymes which have known PDB structure) throughout where the distribution was different, as expected. What we observed in the diseased datasets, therefore, was a significant enrichment in disorder. In other words, it not only confirms the robustness of the tool we used but also our data suggests that in higher organisms disorder is more prevalent leading to increasing interaction complexity.

Additionally, longer stretches of unstructured regions are more prevalent in proteins of “HD dataset”. It is not clear whether it has anything to do with HD pathogenesis. However, increasing length of polyQ stretch in Htt is directly correlated with the age at onset of the disease [35] and intrinsic unstructuredness in the polyQ region [15]. It is also reported that in lower organisms the polyQ length is smaller [36]. A tripartite model correlating the length of protein unstructuredness, its influence on disease outcome and the network complexity, is still elusive.

In conclusion, the fact that “unstructured” proteins are prevalent in “complex” disorders and not necessarily in any kind of genetic disorder, and the observation that they cluster around network “hubs”, may have far reaching consequences in the pursuit of a “specific” solution to these diseases.

## Methods

### Construction of datasets

For analysis, databases were constructed using the following criteria: (A) Proteins involved in HD, PD and AD were retrieved directly from the NCBI's (Http://ww.ncbi.nlm.nih.gov) Entrez Gene database using keywords “Huntington disease Homo sapiens”, “Parkinson disease Homo sapiens” and “Alzheimer disease Homo sapiens”, respectively, for the three diseases. (B) Extensive literature survey was done for reports of proteins interacting with the hubs of the diseases (Huntingtin (Htt) in HD, Amyloid precursor protein (APP) in AD and α-synuclein in PD), primarily determined through high to moderate throughput protein-protein interaction (PPI) or expression studies (e.g., co-immunoprecipitation [37] or microarray analysis [38]) (C) Both the datasets, (A) and (B), were now combined. In case of AD or PD the number of novel hits from step (B) were less, whereas about 115 interactors were incorporated from literature in case of HD (see Table S1 and S2 for references). These enriched datasets were now checked for non-redundancy, any repetition was eliminated, and they were further filtered to remove genetic association results. We realized that the datasets obtained through keyword “text” search carry a finite probability of false positives as well as inadvertent “misses”. In addition, those retrieved through “high-throughput” (genomics or proteomics) studies reportedly contain large number of false positives [39]. Thus, understandably to some extent, the datasets would be “noisy” and biased towards network “hubs” chosen. To avoid this problem, we adopted a stringent filtering criteria where each protein of these datasets were checked to ascertain that their interaction was either physically validated by some other experiment or they had a direct functional implication in the disease pathogenesis as reported in the literature. Some of these studies reported physical interactions of Htt, APP or α-synuclein with mouse proteins. In the dataset we incorporated the human homologues of these proteins, if available. The refined subsets of proteins now had a direct relevance to the diseases and were designated as “HD dataset” (Table S1 and S2), “PD dataset” (Table S3 and S4) and “AD dataset” (Table S5 and S6) respectively. Individual protein sequences were extracted from SWISSPROT database (release 55.0) (http://www.SwissProt.org) using protein IDs.

Along with these datasets, three control datasets were constructed. This first one, “control dataset 1” consisted of 17159 human proteins obtained using SwissProt sequence retrieval system (SRS) by searching the query “[swiss_prot-Organism: homo sapiens*] ! [swiss_prot-Keywords: disease*]”. This control dataset was constructed to check the trend for all human proteins from the SwissProt database that are not annotated to be involved in any disease.

A second control (“control dataset 2”) (Table S7) was constructed which was biased in favor of the conventional wisdom of the “structure-function” paradigm. Initially the dataset consisted of all the enzymes (presumed to have more ordered structure) taken from databases like “Brenda” (Http://www.brenda.uni-koeln.de/) and KEGG, (Http://www.genome.jp/kegg/), involved in various metabolic and biosynthesis pathways like Glycolysis/Gluconeogenesis, TCA cycle, PPP pathway, Starch metabolism, Urea cycle, Fatty acid synthesis, Fatty acid metabolism, Purine metabolism, Pyrimidine metabolism, Bile acid synthesis. Galactose metabolism, Sterol biosynthesis, Nucleotide sugar metabolism, Lysine biosynthesis, Gly-Ser, Thr metabolism, Fructose mannose metabolism, amino sugar metabolism, sphingolipid metabolism, degradation and synthesis of ketone bodies, glutamate metabolism, tyrosine metabolism, histidine metabolism, inositol metabolism, Glycerophospholipid metabolism, Cysteine metabolism, Valine, Leucine, Isoleucine biosynthesis, Phenylalanine metabolism, Alanine metabolism, Valine leucine isoleucine degradation, Arginine proline metabolism, Beta alanine metabolism, Riboflavin metabolism, Lipopolysaccharide biosynthesis, Folate Biosynthesis, Porphyrin metabolism and N-glycan biosynthesis. This raw dataset (consisting of 380 enzymes) was filtered to contain only those enzymes which have known structures submitted to PDB (http://www.rcsb.org). This “control dataset 2” (see Table S7), consisting of 264 metabolic enzymes with known structures, was non-homologous to our disease datasets as these were not the types of proteins usually involved in neurodegenerative processes. We included this control in our analysis to compensate for false positives/negatives.

The third control dataset, “control dataset 3”, consisted of 117 human proteins obtained using SwissProt sequence retrieval system (SRS) by searching the query “[libs = {swiss_prot trembl}-Organism: homo sapiens*] & [libs-Description: breast* & cancer*] ” (see Table S8). This control dataset was constructed to check the trend for the proteins involved in a non-neurodegenerative disease to validate if the involvement of IUP was specific for neurodegeneration or more generally related to diseases.

### Disorder Prediction

Several disorder prediction tools are available to find out disorder regions in proteins [40]–[48]. Several studies [44]–[49] compared the efficacies of these tools and the general conclusion was that performances of FoldIndex and PONDR VL-XT were comparable. FoldIndex uses charge-hydropathy classification originally proposed by Uversky and gives a single index (R) for the entire protein, with a reported accuracy rate of 77% [44]. On the other hand, PONDR-VLXT gives a per residue output based on a neural network prediction with a reported accuracy rate of 72% [44]. We did a random statistical analysis of a few easily available tools [40]–[48] and found out that FoldIndex is definitely one of the better ones (see Text S1). Even though an updated version of PONDR is now available, due to the ease of use in batch through a customized Perl script, we used FoldIndex to classify proteins in terms of unstructuredness. Considering the fact that a large number of false positives/negatives would occur in our constructed datasets, due to conspicuous incompleteness of experimental information, we realized that as predictions were done on the basis of sequence features, use of a single binary classifier might compromise the sensitivity of our prediction. We noted that in the dynamic models of “fuzzy” complexes [2], just a stretch of intrinsic disordered region (IDR) could be sufficient for a protein to behave as an IUP. Hence, occurrence of a stretch of 30 continuous residues in a protein, whether or not FoldIndex classified the protein as an IUP as a whole, was also considered as a metric for IUP in our model. Therefore, we considered a protein to be “unstructured” if (A) it was indexed by FoldIndex (R<0) as an IUP and/or (B) it contained a stretch of 30 consecutive unstructured residues. The unstructured datasets were constructed based on these criteria as described in Table 1.

### Interaction and function

The basis of our analysis was the disease datasets, comprising of unique interactors of disease-gene products, which were manually verified as described before. We searched the interaction database BIOGRID v. 2.0.36 [50] to find out the possible number of interactors, reported till date, of the proteins that are present in the disease datasets. HD being a monogenic disease, huntingtin (*htt*) gene was considered to be the “hub” in the network. For AD and PD, mutations in multiple genes (APP, PS1, PS2) are implicated, while specific allele of APOE4 consistently increase the risk of familial AD (∼5% of total AD incidence). In PD, disease causing mutations at α-synuclein, β-synuclein, PARK2, PARK5 and PARK7 have been identified. However, the genetic reasons behind a large number of familial PD cases are still unknown. Therefore, for these two diseases, the network architecture would have multiple “hubs” with overlaps. To avoid confusions, the two main unambiguously known causative genes i.e., APP and α-synuclein for AD and PD respectively, were considered to construct the respective datasets. Considering that many novel Htt interacting proteins present in the HD dataset obtained from the literature were hardly studied and hence very little or no information was available about their interactions and functions in BIOGRID. Similar were the findings for several AD and PD dataset proteins. On the other hand, for several functionally significant proteins extensive information was already available. There is a definite possibility of biasness during the measurement of the correlation between disorderedness and the number of interactors of the proteins. However, following Haynes et al., we chose ten partners as a cutoff value for a protein designated to be a “hub protein” [24]. But deviating from Haynes et al., we designated all the proteins with less than 10 interactors as “end proteins”. To eliminate any possible biasness in the definition of “hub” and “end” proteins, we varied the cutoff values for “hub” proteins in two consecutive validation analyses and rather than defining a protein with more than 10 interactors as a “hub”, those with more than 5 or 20 interactors in the disease datasets were defined as “hubs” respectively. In each case “end proteins” were defined as those having less than 5 or 20 interactors respectively.

We calculated the percentage of IUPs among the “hub” proteins in the three disease datasets separately, grouped them, calculated the mean and the standard deviation, and defined the value as percentage of IUPs in neurodegenerative disease datasets. To test significant abundance of IUPs among hub proteins Student's t-test was performed and p values were calculated with the aid of “GraphPad QuickCalcs” (Http://www.graphpad. com/quickcalcs/ttest1.cfm?Format=SD). The percentage unstructuredness in End proteins was calculated in a similar way.

### Functional Classification of Unstructured Proteins in Neurodegenerative Diseases

To decipher the range of functions where the IUPs participate, functional annotations were done using PANTHER server (Http://www.pantherdb.org/). PANTHER is a comprehensive database designed to relate protein sequences to functions [51]. Functions were searched for all the proteins in the disease (HD, PD and AD) datasets. All the protein IDs for the three disease-datasets were submitted separately in batch to annotate the biological process in the “PANTHER” annotation tool and the results were tabulated and analyzed. To find significant contribution of any “biological processes” in the disease datasets, the annotations were compared with that for the total human proteome and chi-square tests were performed to calculate the p values. Also to find out significant contribution of unstructuredness in any biological processes, we needed to compare the entire “unstructured” protein population with respect to the “structured” population under each process category and Z-tests were carried out according to Spiegel et al [52].

## Supporting Information

Text S1 Statistical Analysis Prior to Disorder Prediction
(0.01 MB PDF)

Table S1 Huntington's disease Protein Dataset. Proteins that contain ≪30 amino acids residues unstructured at a stretch are tabulated here
(0.01 MB PDF)

Table S2 Huntington's disease Protein Dataset. Proteins that contain ≫30 amino acids residues unstructured at a stretch are tabulated here
(0.02 MB PDF)

Table S3 Parkinson's disease Protein Dataset. Proteins that contain ≪30 amino acids residues unstructured at a stretch are tabulated here
(0.01 MB PDF)

Table S4 Parkinson's disease Protein Dataset. Proteins that contain ≫30 amino acids residues unstructured at a stretch are tabulated here
(0.01 MB PDF)

Table S5 Alzheimer's disease Protein Dataset. Proteins that contain ≪30 amino acids residues unstructured at a stretch are tabulated here
(0.01 MB PDF)

Table S6 Alzheimer's disease Protein Dataset. Proteins that contain ≫30 amino acids residues unstructured at a stretch are tabulated here
(0.02 MB PDF)

Table S7 Control dataset 2 consisting of metabolic enzymes with known structures
(0.01 MB PDF)

Table S8 Control dataset 3 consisting of proteins involved in Breast Cancer
(0.08 MB PDF)
