# Supplementary material for: The Role of Intrinsically Unstructured Proteins in Neurodegenerative Diseases
Source: PLoS One. 2009 May 15;4(5):e5566. doi: 10.1371/journal.pone.0005566 (PMC2679209; doi:10.1371/journal.pone.0005566)
Supplement: Text S1 — Statistical Analysis Prior to Disorder Prediction (0.01 MB PDF) [file pone.0005566.s001.pdf]

Using a dataset of 39 known experimentally obtained disordered proteins/domains, they found 77% of the proteins were correctly predicted by FoldIndex, 72% by PONDR VL-XT and 20% by GlobPlot respectively. We used the same dataset of 39 unstructured proteins for disorder prediction by IUPRED, DisEMBL and RONN to check the accuracy of predictions by these tools. RONN and IUPRED predicted 58.33% correctly and DisEMBL only 19.4%. Further, a dataset of 46 proteins was compiled from a search of HD proteins from the NCBI's Entrez Gene database by giving the query "Huntington disease Homo sapiens". All of these proteins were checked for disorderedness using all the six tools stated above. Of these, 34 and 36 proteins were predicted as unstructured by FoldIndex and PONDR Vl-XT respectively. Globplot, DisEMBL, IUPRED and RONN predicted only 18, 19, 14, and 18 of the total 46 proteins as disordered, respectively. Owing to these findings, GlobPlot, DisEMBL, IUPRED and RONN, having lesser accuracy level, were not used any further in our analysis. Hence, FoldIndex, with a reported accuracy rate of 77% was used all through, for all the disease and control datasets.
