# Supplementary material for: The Role of Intrinsically Unstructured Proteins in Neurodegenerative Diseases
Source: PLoS One. 2009 May 15;4(5):e5566. doi: 10.1371/journal.pone.0005566 (PMC2679209; doi:10.1371/journal.pone.0005566)
Supplement: Table S1 — Huntington's disease Protein Dataset. Proteins that contain ≪30 amino acids residues unstructured at a stretch are tabulated here (0.01 MB PDF) [file pone.0005566.s002.pdf]

	Official Symbo	Numb	NCBI ID	Official full name	% uns	Leng	Reference
1	MT-CO2	No Bl	NP_536846.1	mitochondrially encoded cytochrome c oxidase II	0	0	Kaltenbach et al., 2007, Plos Genet., 3:e82.
2	MT-CO3	No Bl	NP_536849.1	mitochondrially encoded cytochrome c oxidase III	0	0	Kaltenbach et al., 2007, Plos Genet., 3:e82.
3	COX5B	4	NP_001853.2	cytochrome c oxidase subunit Vb	0	0	Kaltenbach et al., 2007, Plos Genet., 3:e82.
4	COX6C	1	NP_004365.1	cytochrome c oxidase subunit VIc	0	0	Kaltenbach et al., 2007, Plos Genet., 3:e82.
5	COX5A	1	NP_004246.1	cytochrome c oxidase subunit Va	18	14	Kaltenbach et al., 2007, Plos Genet., 3:e82.
6	DLD	1	NP_000099.2	dihydrolipoamide dehydrogenase	0	0	Kaltenbach et al., 2007, Plos Genet., 3:e82.
7	LDHA	4	NP_005557.1	lactate dehydrogenase A	0	0	Kaltenbach et al., 2007, Plos Genet., 3:e82.
8	LDHB	2	NP_002291.1	lactate dehydrogenase B	1.49	5	Kaltenbach et al., 2007, Plos Genet., 3:e82.
9	CCT6A	1	NP_001009186.1	chaperonin containing TCP1, subunit 6A	0	0	Kaltenbach et al., 2007, Plos Genet., 3:e82.
10	UQCRC2	5	NP_003357.2	ubiquinol-cytochrome c reductase core protein II	0	0	Kaltenbach et al., 2007, Plos Genet., 3:e82.
11	PKM2	4	NP_002645.3	pyruvate kinase, muscle	1.3	7	Kaltenbach et al., 2007, Plos Genet., 3:e82.
12	ENO1	3	NP_001419.1	enolase 1	4.37	19	Kaltenbach et al., 2007, Plos Genet., 3:e82.
13	TPP1	0	NP_000382.3	tripeptidyl peptidase I	7.1	15	Kaltenbach et al., 2007, Plos Genet., 3:e82.
14	CD59	16	NP_000602.1	CD59	9.3	12	Kaltenbach et al., 2007, Plos Genet., 3:e82.
15	ENO3	0	NP_001967.1	enolase 3	10.4	27	Kaltenbach et al., 2007, Plos Genet., 3:e82.
16	VDAC1	35	NP_003365.1	voltage-dependent anion channel 1	17.7	27	Kaltenbach et al., 2007, Plos Genet., 3:e82.
17	GAPDH	26	NP_002037.2	glyceraldehyde-3-phosphate dehydrogenase	0	0	Burke et al., 1996, Nat Med. 2:347-50
18	PFN2	18	NP_002619.1	profilin 2	0	0	http://hdbase.org
19	CTBP1	82	NP_001012632.1	C-terminal binding protein 1	1.59	7	Kegel et al., 2002, J Biol Chem. 277:7466-76.
20	ECH1	0	NP_001389.2	enoyl Coenzyme A hydratase 1, peroxisomal	6.09	15	http://hdbase.org
21	HYPE	1	NP_009007.2	Huntingtin interacting protein E	6.76	26	Faber et al., 1998, Hum Mol Genet. 7:1463-1474.
22	F8A1/HAP40	1	NP_036283.2	coagulation factor VIII-associated (intronic transcript)	12.3	27	Peters et al., 2001., J Biol Chem., 276:3188-3194.
23	PDE1A	2	NP_001003683.1	phosphodiesterase 1A, calmodulin-dependent	23.2	27	http://hdbase.org
24	GRAP	6	NP_006604.1	GRB2-related adaptor protein	29	26	http://hdbase.org
25	EEF1A1	22	NP_001393.1	eukaryotic translation elongation factor 1 alpha 1	8.6	27	Mitusi et al., 2002, J Neurosci., 22:9267-77.
26	NEGR1	0	NP_776169.2	neuronal growth regulator 1	5	12	Kaltenbach et al., 2007, Plos Genet., 3:e82.
27	GPM6A	0	NP_005268.1	glycoprotein M6A	1	28	Kaltenbach et al., 2007, Plos Genet., 3:e82.
