# Supplementary material for: The Role of Intrinsically Unstructured Proteins in Neurodegenerative Diseases
Source: PLoS One. 2009 May 15;4(5):e5566. doi: 10.1371/journal.pone.0005566 (PMC2679209; doi:10.1371/journal.pone.0005566)
Supplement: Table S2 — Huntington's disease Protein Dataset. Proteins that contain ≫30 amino acids residues unstructured at a stretch are tabulated here (0.02 MB PDF) [file pone.0005566.s003.pdf]

	Official Symb	Num	NCBI ID	Official full name	% unstr	Lengt	Reference
1	COX6B1	1	NP_001854.1	cytochrome c oxidase subunit Vib polypeptide 1 (ubiquitous)	68.6	59	Kaltenbach et al., 2007, Plos Genet., 3:e82.
2	CLTC	49	NP_004850.1	clathrin, heavy chain (Hc)	7.46	36	Kaltenbach et al., 2007, Plos Genet., 3:e82.
3	GRIK5	7	NP_002079.3	glutamate receptor, ionotropic, kainate 5	12.19	36	Kaltenbach et al., 2007, Plos Genet., 3:e82.
4	DLST	2	NP_001924.2	dihydrolipoamide S-succinyltransferase	12.36	60	Kaltenbach et al., 2007, Plos Genet., 3:e82.
5	HIP1	24	NP_005329.3	huntingtin interacting protein 1	44.75	199	Wanker et al., 1997, HMG, 6, 487-495.
6	HIP2 / HYPG	9	NP_005330.1	huntingtin interacting protein 2	29.64	49	Kalchman et al., 1996, JBC, 271,19385-19394.
7	HIP14/ZDHHC	6	NP_056151.2	zinc finger, DHHC-type containing 17	15.98	76	Singaraja et al., 2002, HMG, 11, 2815-2828.
8	HIP4/CBS	9	NP_000062.1	cystathionine-beta-synthase	20.18	62	Boutell et al. 1998, HMG, 7, 371- 378
9	HAP1	29	NP_001073339.1	huntingtin-associated protein 1 (neuroan 1)	63.48	133	Li et al. Nature, 1995, 378, 398-402.
10	HIP7/ FIP2/HY	10	NP_001008212.1	optineurin	86.65	152	Faber et al., 1998, HMG, 7, 1463-1474.
11	HIP9/ HYPJ/A	11	NP_036437.1	adaptor-related protein complex 2, alpha 2 subunit	9.58	48	Faber et al., 1998, HMG, 7, 1463-1474.
12	VDAC2	8	NP_003366.2	voltage-dependent anion channel 2	17.57	32	Kaltenbach et al., 2007, Plos Genet., 3:e82.
13	DYNC1H1	7	NP_001367.2	dynein, cytoplasmic 1, heavy chain 1	19.45	89	Kaltenbach et al., 2007, Plos Genet., 3:e82.
14	SYP	7	NP_003170.1	synaptophysin	22.36	70	Kaltenbach et al., 2007, Plos Genet., 3:e82.
15	CYC1	2	NP_001907.2	cytochrome c-1	24.3	40	Kaltenbach et al., 2007, Plos Genet., 3:e82.
16	CACNA2D1	0	NP_000713.2	calcium channel, voltage-dependent, alpha 2/delta subunit 1	25.9	52	Kaltenbach et al., 2007, Plos Genet., 3:e82.
17	COX6A1	0	NP_004364.2	cytochrome c oxidase subunit VIa polypeptide 1 precursor	30	33	Kaltenbach et al., 2007, Plos Genet., 3:e82.
18	ITPR1	36	NP_001093422.1	inositol 1,4,5-triphosphate receptor, type 1	30.39	90	Kaltenbach et al., 2007, Plos Genet., 3:e82.
19	CKM	3	NP_001815.2	creatine kinase	36.48	60	Kaltenbach et al., 2007, Plos Genet., 3:e82.
20	SYN1	17	NP_008881.2	synapsin I	46.24	249	Kaltenbach et al., 2007, Plos Genet., 3:e82.
21	PRNP	11	NP_000302.1	prion protein (p27-30)	58.89	81	Kaltenbach et al., 2007, Plos Genet., 3:e82.
22	STX1A	69	NP_004594.1	syntaxin 1A (brain)	59.02	164	Kaltenbach et al., 2007, Plos Genet., 3:e82.
23	SNAP25	35	NP_003072.2	synaptosomal-associated protein, 25kDa	83.98	65	Kaltenbach et al., 2007, Plos Genet., 3:e82.
24	CASP2	30	NP_116764.2	caspase 2	13.7	57	Hermel et al., 2004, Cell. Death. Differ., 11:424-438.
25	TUBB	34	NP_821133.1	tubulin, beta	13.96	58	Hoffner et al., 2002, J. Cell. Science., 115:941-948.
26	TAF4	9	NP_003176.2	TAF4 RNA polymerase II, TATA box binding protein (TBP)-asso	16.3	117	Dunah at al., 2002, Science, 296:2238-43.
27	CRMP1	2	NP_001014809.1	collapsin response mediator protein 1	19.93	75	http://hdbase.org
28	SYMPK	3	NP_004810.2	symplekin	20.48	73	Faber et al., 1998, HMG, 7:1463-1474.
29	TGM2	31	NP_004604.2	transglutaminase 2	21.54	58	Chun et al., 2001, Neurobiol. Dis., 8:391-404.
30	CASP6	7	NP_001217.2	caspase 6	22.52	66	Hermel et al., 2004, Cell. Death. Differ., 11:424-438.
31	SH3GLB1	14	NP_057093.1	SH3-domain GRB2-like endophilin B1	22.73	51	Modregger et al., 2003, J. Bio. Chem., 278:4160-4167
32	TPH1	3	NP_004170.1	tryptophan hydroxylase 1 (tryptophan 5-monooxygenase)	23.42	59	Yohrling et al., 2002, J Neurochem. 82:1416-23.
33	EGFR	183	NP_005219.2	epidermal growth factor receptor (erythroblastic leukemia viral (	23.71	131	Liu et al., 1997, J. Biol Chem., 272:8121-8124.
34	TBP	98	NP_003185.1	TATA box binding protein	24.77	73	Schaffar et al., 2004, Mol. Cell., 15:95-105.
35	CASP7	28	NP_001218.1	caspase 7	25.4	77	Hermel et al., 2004, Cell. Death. Differ., 11:424-438.
36	SHC1	190	NP_003020.2	SHC (Src homology 2 domain containing) transforming protein 1	26.24	55	http://hdbase.org
37	RASA1	85	NP_002881.1	RAS p21 protein activator (GTPase activating protein) 1	26.83	103	Liu et al., 1997, J. Biol Chem., 272:8121-8124.
38	CASP3	80	NP_004337.2	caspase 3	27.79	33	Zhang et al., 2006, EMBO J., 25:5896-5906.
39	TUBG1	32	NP_001061.2	tubulin, gamma 1	28.6	71	Hoffner et al., 2002, J. Cell. Science., 115:941-948.
40	NFKB1	108	NP_003989.2	nuclear factor of kappa light polypeptide gene enhancer in B-ce	29	80	Takano et al., 2002, BMC Neuroscience., 3:1-13.
41	PCAF	44	NP_003875.3	p300/CBP-associated factor	30.8	63	Steffan et al., 2001, Nature., 413:739-43.
42	XRCC6/KU70	78	NP_001460.1	X-ray repair complementing defective repair in Chinese hamste	32.34	59	Li et al., 2007, Cell Death Differ. 14:2058-67.
43	MAGEA3/HIP8	0	NP_005353.1	melanoma antigen family A, 3	35.98	61	Faber et al., 1998, HMG, 7:1463-1474.
44	DLG4/PSD95	209	NP_001356.1	postsynaptic density protein 95	37.98	75	Sun et al., 2001, J. Biol. Chem., 276:24713-24718.
45	CREB1	36	NP_004370.1	cAMP responsive element binding protein 1	38.4	68	Shimohata et al., 2000, Nat Genet. 26:29–36.
46	AKT1	77	NP_001014431.1	v-akt murine thymoma viral oncogene homolog 1	39.16	56	Humbert et al., 2002. Dev. Cell., 2:831-837.

47 HIP1R	12	NP_003950.1	huntingtin interacting protein 1 related	40.07	107	Okano et al., 2003, Brain Res. 967:210-25.
48 MED31/CG-12	6	NP_057144.1	mediator complex subunit 31	40.45	35	Goehler et al., 2004, Mol. Cell., 15: 853-865.
49 SP1	99	NP_612482.2	Sp1 transcription factor	40.5	148	Dunah at al., 2002, Science, 296:2238-43.
50 GIT1	38	NP_001078923.1	G protein-coupled receptor kinase interactor 1	42.08	82	Goehler et al., 2004, Mol. Cell., 15: 853-865.
51 CASP8	59	NP_001073593.1	caspase 8	42.79	70	Sanchez et al., 1999, Neuron., 22, 623-633.
52 HSPA4/HSP7(	21	NP_002145.3	heat shock 70kDa protein 4	43.45	172	Cornett et al., 2005, Nat. Genet., 37: 198-204.
53 DNM3	2	NP_056384.2	dynamin 3	43.66	344	Kaltenbach et al., 2007, Plos Genet., 3:e82.
54 MAP3K10	21	NP_002437.2	mitogen-activated protein kinase kinase kinase 10	44.86	112	Liu et al., 2000, J. Biol Chem., 275:19035-19040.
55 SAP30	16	NP_003855.1	Sin3A-associated protein, 30kDa	47.72	85	http://hdbase.org
56 CREBBP	174	NP_001073315.1	CREB binding protein (Rubinstein-Taybi syndrome)	47.9	142	Nucifora et al., 2001, Science, 291:2423-2438
57 IFT57/HIPPI	4	NP_060480.1	intraflagellar transport 57 homolog (Chlamydomonas)	48.48	97	Gervais et al., 2002, Nat. Cell. Biol.,4:95-105
58 SH3GL3	11	NP_003018.2	SH3-domain GRB2-like 3	50	54	Sittler et al., 1998, Mol. Cell., 2:427-436.
59 CHD3	17	NP_001005271.2	chromodomain helicase DNA binding protein 3	51.55	224	http://hdbase.org
60 SIN3A	73	NP_056292.1	SIN3 homolog A, transcription regulator (yeast)	54.2	165	Steffan et al., 2000, PNAS., 97:6763-6768.
61 MTSS1	0	NP_055566.3	metastasis suppressor 1	55.49	122	http://hdbase.org
62 EIF2AK2	54	NP_002750.1	eukaryotic translation initiation factor 2-alpha kinase 2	55.53	89	Peel et al., 2001, Hum. Mol. Genet., 10:1531-1538.
63 DNM1L/DRP1	5	NP_005681.1	dynamin 1-like	57.57	75	Qin et al., 2004, J Neurosci., 24:269-81
64 DNALI1/P28	0	NP_003453.2	dynein, axonemal, light intermediate chain 1	58.9	77	Goehler et al., 2004, Mol. Cell., 15: 853-865.
65 DNAJB1/HSP4	17	NP_006136.1	DnaJ (Hsp40) homolog, subfamily B, member 1	59.7	85	Jana et al., 2000, Hum. Mol. Genet., 2009-2018.
66 PTPN11	149	NP_002825.3	protein tyrosine phosphatase, non-receptor type 11 (Noonan sy	59.79	85	http://hdbase.org
67 TP53	380	NP_000537.3	tumor protein p53 (Li-Fraumeni syndrome)	62.08	128	Bae et al., 2005, neuron., 47:29-41.
68 GRB2	410	NP_002077.1	growth factor receptor-bound protein 2	63.83	161	Liu et al., 1997, J. Biol Chem., 272:8121-8124.
69 TCERG1/CA1	2	NP_001035095.1	transcription elongation regulator 1	66.39	346	Holbert et al., 2001, PNAS, 98, 1811-1816.
70 NCOR1	72	NP_006302.2	nuclear receptor co-repressor 1	66.51	268	Boutell et al., 1999, Hum. Mol. Genet., 8:1647-1655.
71 PRPF40A/HYI	37	NP_060362.3	PRP40 pre-mRNA processing factor 40 homolog A (S. cerevisia	67.92	297	Faber et al., 1998, HMG, 7:1463-1474.
72 SETD2/HYPB	3	NP_054878.3	SET domain containing 2	67.97	526	Faber et al., 1998, HMG, 7:1463-1474.
73 PRPF40B/HYI	2	NP_001026868.1	PRP40 pre-mRNA processing factor 40 homolog B (S. cerevisia	73	281	Faber et al., 1998, HMG, 7:1463-1474.
74 MBD1/PCM1	7	NP_002375.1	methyl-CpG binding domain protein 1	75.03	313	Engelender et al., 1997, Hum Mol Genet. 6:2205-12.
75 REST	6	NP_005603.2	RE1-silencing transcription factor	79.12	588	Zuccato et al., 2003, Nat Genet., 35:76-83.
76 TPR	7	NP_003283.2	translocated promoter region (to activated MET oncogene)	79.18	302	Cornett et al., 2005, Nat. Genet., 37: 198-204.
77 FEZ1	7	NP_005094.1	fasciculation and elongation protein zeta 1 (zygin I)	81.37	180	Kaltenbach et al., 2007, Plos Genet., 3:e82.
78 HYPK	1	NP_057484.3	Huntingtin interacting protein K	81.71	105	Faber et al., 1998, HMG, 7:1463-1474.
79 TRIP10	24	NP_004231.1	thyroid hormone receptor interactor 10	82.02	130	Holbert et al., 2003, PNAS., 100:2712-2717
80 CXorf27/HYPI	0	NP_036406.1	chromosome X open reading frame 27	84.6	58	Faber et al., 1998, HMG, 7:1463-1474.
81 PACSIN1	19	NP_065855.1	protein kinase C and casein kinase substrate in neurons 1	91.21	232	Modregger et al., 2002, Hum. Mol. Genet., 11:2547-2558.
82 PQBP1	10	NP_001027553.1	polyglutamine binding protein 1	93.58	188	Waragai et al., 1999, Hum Mol Genet., 8:977-87.
83 SUMO1	70	NP_001005781.1	SMT3 suppressor of mif two 3 homolog 1 (S. cerevisiae)	98	41	Steffan et al., 2004, Science., 304:100-104.
84 GRIN2A	39	NP_000824.1	glutamate receptor, ionotropic, N-methyl D-aspartate 2A	42.55	228	Waxman et al., 2005, Neuroscientist., 11:37-49
85 RFWD2/COP1	5	NP_001001740.1	ring finger and WD repeat domain 2	58	97	Wang et al., 2005, J Neurosci., 25:11645-54.
86 BDNF	7	NP_001700.2	brain-derived neurotrophic factor	47.7	45	Luthi-Carter et al., 2002, Hum. Mol. Genet., 11:1927-1937.
87 EP300/P300	144	NP_001420.2	E1A binding protein p300	51.78	155	http://hdbase.org
88 CALML3/CLP	3	NP_005176.1	calmodulin-like 3	75	48	Bao et al., 1996., PNAS, 93:5037-5042.
89 SNAP91/CALI	5	NP_055656.1	synaptosomal-associated protein, 91kDa homolog (mouse)	97.9	97	Bao et al., 1996., PNAS, 93:5037-5042.
90 KALRN/HAPIF	1	NP_001019831.2	kalirin, RhoGEF kinase	39.14	63	Colomer at al., 1997, Hum Mol Genet. 6:1519-25
91 CALML6	0	NP_619650.2	calmodulin-like 6	74.5	39	Bao et al., 1996., PNAS, 93:5037-5042.
92 RIPK2	30	NP_003812.1	receptor-interacting serine-threonine kinase 2	14.4	60	Wang et al., 2005, J Neurosci., 25:11645-54.
93 HD	71	NP_002102.4	huntingtin (Huntington disease)	14.91	129	HDCRG, 1993, Cell., 72:971–983.
94 ZNF395/HDBF	0	NP_061130.1	zinc finger protein 395	35.86	61	Tanaka at al., 2004, J Biol. Chem., 279:7275-86.
95 SLC2A4RG/HI	0	NP_064446.2	SLC2A4 regulator	34.1	56	Tanaka at al., 2004, J Biol. Chem., 279:7275-86.

96	HIP6/HYPF/PS	8	NP_002803.2	proteasome (prosome, macropain) 26S subunit, non-ATPase, 8	25.1	61	Faber et al., 1998, Hum Mol Genet. 7:1463-1474.
97	DNAJA3	19	NP_005138.2	DnaJ (Hsp40) homolog, subfamily A, member 3	42	80	Kaltenbach et al., 2007, Plos Genet., 3:e82.
98	DNAJA5	0	NP_001012339.2	DnaJ homology subfamily A member 5	96.9	392	Kaltenbach et al., 2007, Plos Genet., 3:e82.
99	DNAJC4	0	NP_005519.2	DnaJ (Hsp40) homolog, subfamily C, member 4	74.6	86	Kaltenbach et al., 2007, Plos Genet., 3:e82.
##	DNAJC11	0	NP_060668.1	DnaJ (Hsp40) homolog, subfamily C, member 11	32.9	93	Kaltenbach et al., 2007, Plos Genet., 3:e82.
##	DCTN2	6	NP_006391.1	dynactin 2 (p50)	38.4	57	Kaltenbach et al., 2007, Plos Genet., 3:e82.
##	SP3	12	NP_001017371.1	Sp3 transcription factor	34.6	174	Kaltenbach et al., 2007, Plos Genet., 3:e82.
##	DCTN1	41	NP_004073.2	dynactin 1 (p150, glued homolog, Drosophila)	43.6	344	http://hdbase.org
##	BAG1	42	NP_004314	BCL2-associated athanogene	49.2	156	Jana et al., 2005, Neurosci Lett., 378:171-175.
##	RAB8A	12	NP_005361.2	RAB8A, member RAS oncogene family	31.8	39	Hattula et al., 2000, Curr Biol., 10:1603-1606.
##	ADD3	0	NP_001112.2	adducin 3 (gamma)	47.9	168	Kaltenbach et al., 2007, Plos Genet., 3:e82.
##	GFAP	24	NP_002046.1	glial fibrillary acidic protein	65.5	107	Kaltenbach et al., 2007, Plos Genet., 3:e82.
##	KIAA1229	0	NP_055906.2	KIAA0423	30.6	87	Kaltenbach et al., 2007, Plos Genet., 3:e82.
##	PPL	12	NP_002696.3	periplakin	82.8	222	Kaltenbach et al., 2007, Plos Genet., 3:e82.
##	SEPT7/CDC3	6	NP_001011553.2	septin 7	48.05	141	Kaltenbach et al., 2007, Plos Genet., 3:e82.
##	SORBS1	22	NP_001030126.1	sorbin and SH3 domain containing 1	57.7	317	Kaltenbach et al., 2007, Plos Genet., 3:e82.
##	GNAZ	19	NP_002064.1	guanine nucleotide binding protein (G protein)	24.22	40	Kaltenbach et al., 2007, Plos Genet., 3:e82.
##	YWHAG	85	NP_036611.2	tyrosine 3-monooxygenase/tryptophan 5-monooxygenase activa	46.15	41	Kaltenbach et al., 2007, Plos Genet., 3:e82.
##	YWHAB	120	NP_003395.1	tyrosine 3-monooxygenase/tryptophan 5-monooxygenase activa	59.3	52	Kaltenbach et al., 2007, Plos Genet., 3:e82.
##	NAPB	0	NP_071363.1	N-ethylmaleimide-sensitive factor attachment protein, beta	24.8	33	Kaltenbach et al., 2007, Plos Genet., 3:e82.
##	USP9X	5	NP_001034679.2	ubiquitin specific peptidase 9, X-linked	23.3	109	Kaltenbach et al., 2007, Plos Genet., 3:e82.
##	MEF2D	18	NP_005911.1	myocyte enhancer factor 2D	38.1	67	Kaltenbach et al., 2007, Plos Genet., 3:e82.
##	PPARG	54	NP_005028.4	peroxisome proliferator-activated receptor gamma	20.5	50	Kaltenbach et al., 2007, Plos Genet., 3:e82.
##	ZNF91	0	NP_003421.2	zinc finger protein 91	89.4	549	Kaltenbach et al., 2007, Plos Genet., 3:e82.
##	NDUFB10	1	NP_004996.1	NADH dehydrogenase (ubiquinone) 1 beta subcomplex, 9, 22kD	100	179	Kaltenbach et al., 2007, Plos Genet., 3:e82.
