# Supplementary material for: The Role of Intrinsically Unstructured Proteins in Neurodegenerative Diseases
Source: PLoS One. 2009 May 15;4(5):e5566. doi: 10.1371/journal.pone.0005566 (PMC2679209; doi:10.1371/journal.pone.0005566)
Supplement: Table S3 — Parkinson's disease Protein Dataset. Proteins that contain ≪30 amino acids residues unstructured at a stretch are tabulated here (0.01 MB PDF) [file pone.0005566.s004.pdf]

	Official Symbol	Numb	NCBI ID	Official full name	% unstr	Length	Reference
1	TOR1A/DYT1	5	NP_000104.1	torsin family 1, member A (torsin A)	8.4	28	McLean et al., 2002, J Neurochem., 83:846-54.
2	UBE2L6/UBCH8	17	NP_004214.1	ubiquitin-conjugating enzyme E2L 6	0	0	Lee et al., 2008, Hum Mol Genet. 17:906-17.
3	CYP2D6	0	NP_000097.2	cytochrome P450, family 2, subfamily D, polypeptide 6	5.4	27	Duric et al., 2007, Vojnosanit Pregl. 64:25-30.
4	GSTA4	2	NP_001503.1	glutathione S-transferase A4	0	0	Patel et al., 2006, Brain Res., 1081:9-18.
5	NQO1	2	NP_000894.1	NAD(P)H dehydrogenase, quinone 1	9.8	27	Zafar et al., 2006, Toxicol Lett., 166:261-7.
6	HTR6	2	NP_000862.1	5-hydroxytryptamine (serotonin) receptor 6	1.8	21	Cao et al., 2007, Neurosci Bull., 23:315-322.
7	UBE2L3/UBCH7/AMIG	37	NP_003338.1	ubiquitin-conjugating enzyme E2L 3	5.7	17	Shimura et al., 2001, Science., 293:263-9.
8	IL8	11	NP_000575.1	interleukin 8	0	0	Ross et al., 2004, Hum Immunol., 65:340-6.
9	FADD	81	NP_003815.1	Fas (TNFRSF6)-associated via death domain	13.4	28	Hartmann et al., 2002, Neurology., 58:308-10.
10	CNR1/CB1	5	NP_057167.2	cannabinoid receptor 1 (brain)	5.2	25	González et al., 2006 , Brain Res., 1073-1074:209-19.
11	GAD2	2	NP_000809.1	glutamate decarboxylase 2 (pancreatic islets and brain, 65k	4.7	18	Luo et al., 2002, Science., 298:425-9.
12	SIAH1	60	NP_001006611.1	seven in absentia homolog 1 (Drosophila)	3.5	10	Lee et al., 2008, Hum Mol Genet. 17:906-17.
13	SLC18A2/VMAT2	0	NP_003045.2	solute carrier family 18 (vesicular monoamine), member 2	7	28	Chen et al., 2007, J Neurochem., [Epub ahead of print]
14	TH/TYH	3	NP_000351.2	tyrosine hydroxylase	14.4	25	Alerte et al., 2008, Neurosci Lett. [Epub ahead of print]
15	PINK1/PARK6	0	NP_115785.1	PTEN induced putative kinase 1	0	0	Exner et al., 2007, J Neurosci., 27:12413-8.
16	PARK7	7	NP_009193.2	Parkinson disease (autosomal recessive, early onset) 7	0	0	Maita et al., 2007, Neurosci Lett., [Epub ahead of print]
