# Supplementary material for: The Role of Intrinsically Unstructured Proteins in Neurodegenerative Diseases
Source: PLoS One. 2009 May 15;4(5):e5566. doi: 10.1371/journal.pone.0005566 (PMC2679209; doi:10.1371/journal.pone.0005566)
Supplement: Table S4 — Parkinson's disease Protein Dataset. Proteins that contain ≫30 amino acids residues unstructured at a stretch are tabulated here (0.01 MB PDF) [file pone.0005566.s005.pdf]

	Official Symbol	Num	NCBI ID	Official full name	% unstr	Lengt	Reference
1	SLC6A3	8	NP_001035.1	solute carrier family 6 (neurotransmitter transporter, dopamine), mer	6.45	34	Kelada et al., 2006, Hum Mol Genet., 15:3055-62.
2	TUBA1A	No B	NP_006000.2	tubulin, alpha 1a	10.86	49	Alim et al., 2004, J Alzheimers Dis. 6:435-42
3	UCHL1/PARK5	10	NP_004172.2	ubiquitin carboxyl-terminal esterase L1 (ubiquitin thiolesterase)	18.8	35	Wakabayashi et al., 2007, Brain Nerve., 59:851-64.
4	MAPK1	109	NP_002736.3	mitogen-activated protein kinase 1	19	69	Iwata et al., 2001, J Neurochem., 77:239-52.
5	ELK1	17	NP_005220.1	ELK1, member of ETS oncogene family	21.02	43	Iwata et al., 2001, J Neurochem., 77:239-52.
6	PARK2	35	NP_004553.1	Parkinson disease (autosomal recessive, juvenile) 2, parkin	23	38	Wakabayashi et al., 2007, Brain Nerve., 59:851-64.
7	PLD1	26	NP_002653.1	phospholipase D1, phosphatidylcholine-specific	29.32	81	Narayanan et al., 2005 , Biochemistry., 44:462-70.
8	AKT1	79	NP_001014431.1	v-akt murine thymoma viral oncogene homolog 1	39.1	56	Hashimoto et al., 2004, J Biol Chem., 279:23622-9.
9	BCL2L1/BCLX	115	NP_001182.1	BCL2-like 1	30.9	60	Hartmann et al., 2002, Neurobiol Dis. 10:28-32.
10	SNCB	5	NP_001001502.1	synuclein, beta	32.8	44	Snyder et al., 2005, J Biol Chem., 280:7562-9.
11	FYN/SLK	196	NP_002028.1	FYN oncogene related to SRC, FGR, YES	36.94	56	Ellis et al., 2001, J Biol Chem. 276:3879-84.
12	SQSTM1/p60/p62	31	NP_003891.1	sequestosome 1	50.22	121	Nakaso et al., 2004, Brain Res., 1012:42-51.
13	SNCAIP	12	NP_005451.2	synuclein, alpha interacting protein (synphilin)	61.8	176	Humbert et al., 2007, Neurobiol Dis., 26:681-7.
14	CYCS	35	NP_061820.1	cytochrome c, somatic	61.9	56	Wang et al., 2007, Neurobiol Dis., 28:216-26.
15	MAPK8IP1	15	NP_005447.1	mitogen-activated protein kinase 8 interacting protein 1	62.86	274	Xia et al., 2001, Proc Natl Acad Sci U S A., 98:10433-8.
16	MAP1B/MAP5	16	NP_005900.2	microtubule-associated protein 1B	65.88	487	Jensen et al., 2000, J Biol Chem., 275:21500-7.
17	MAPT/TAU	21	NP_005901.2	microtubule-associated protein tau	80	171	Wakabayashi et al., 2007, Brain Nerve., 59:851-64.
18	CALM1	91	NP_008819.1	calmodulin 1 (phosphorylase kinase, delta)	97.9	97	Martinez et al., 2003, J Biol Chem. 278:17379-87.
19	BAD	52	NP_004313.1	BCL2-antagonist of cell death	100	168	Kaul et al., 2005, Brain Res Mol Brain Res., 139:137-52.
20	SNCA	42	NP_000336.1	synuclein, alpha (non A4 component of amyloid precursor)	31.4	44	Thomas et al., 2007, Hum Mol Genet., 16 Spec No. 2:R183-94.
21	ACHE	6	NP_000656.1	acetylcholinesterase (Yt blood group)	18.4	50	Ruberq et al., 1986, Brain Res. 1;362:83-91.
22	CP	5	NP_000087.1	ceruloplasmin (ferroxidase)	9.5	102	Hochstrasser et al., 2005, FASEB J. 19:1851-3.
23	GCH1	2	NP_000152.1	GTP cyclohydrolase 1 (dopa-responsive dystonia)	31.2	78	Duan et al., 2005, Brain Res Brain Res Protoc. 16:37-43.
24	ABCB1/MDR1	0	NP_000918.2	ATP-binding cassette, sub-family B (MDR/TAP), member 1	11	50	Drozdzik et al., 2003, Pharmacogenetics. 1
25	UBQLN1	41	NP_038466.2	ubiquilin 1	8.3	49	Mah et al., 2000, J Cell Biol., 151(4):847-62.
26	PARP1	40	NP_001609.2	poly (ADP-ribose) polymerase family, member 1	8.2	84	Outeiro et al., 2007, Biochem Biophys Res Commun., 357:596-602.
27	LPO	0	NP_006142.1	lactoperoxidase	27.6	58	Everse et al., 2004, Free Radic Biol Med. 37:839-49.
28	GPR37	5	NP_005293.1	G protein-coupled receptor 37 (endothelin receptor type B-like)	8.8	54	Marazziti et al., 2004, Proc Natl Acad Sci U S A., 101:10189-94.
29	SEPT4/MART	6	NP_004565.1	septin 4	60	130	Ihara et al., 2003 , J Biol Chem., 278:24095-102.
30	DBH	2	NP_000778.2	dopamine beta-hydroxylase (dopamine beta-monooxygenase)	5.4	33	Healy et al., 2004, Ann Neurol., 55:443-6.
31	HSP90AA1/HSP90	121	NP_001017963.2	heat shock protein 90kDa alpha (cytosolic), class A member 1	31.04	131	Weihofen et al., 2007, Hum Mol Genet., [Epub ahead of print]
32	NRG1	14	NP_004486.2	neuregulin 1	18.6	119	Zhang et al., 2004, J Neurochem., 91:1358-68.
33	ESR1	154	NP_000116.2	estrogen receptor 1	28.2	86	Liu et al., 2005, J Neurosci Res. 81:653-65.
34	ESR2	44	NP_001035365.1	estrogen receptor 2 (ER beta)	19.6	74	Liu et al., 2005, J Neurosci Res. 81:653-65.
35	UBE1	18	NP_003325.2	ubiquitin-activating enzyme E1	3.5	38	Fornai et al., 2004, J Neurochem. 88(1):114-23.
36	IL1B	5	NP_000567.1	interleukin 1, beta	13.3	36	Koprich et al., 2008 , J Neuroinflammation. 5:8
37	NR4A2/NOT	10	NP_006177.1	nuclear receptor subfamily 4, group A, member 2	10.3	62	Le et al., 2003, Nat Genet. 33:85-9.
38	RNF19A/DORFIN	9	NP_056250.3	ring finger protein 19A	13.7	115	Hishikawa et al., 2003, Am J Pathol., 163:609-19.
39	GAD1	4	NP_000808.2	glutamate decarboxylase 1 (brain, 67kDa)	17.3	40	Stephenson et al., 2005, Neurobiol Dis. 20:347-59.
40	PDYN/PENKB	0	NP_077722.1	prodynorphin	51.9	132	Bäckman et al., J Neurosci Res. 2007 Mar;85(4):798-804.
41	BDNF	7	NP_001700.2	brain-derived neurotrophic factor	29.5	73	Fumagalli et al., 2006, Pharmacogenomics J. 6:95-104.
42	PACRG/GLUP	0	NP_001073847.1	PARK2 co-regulated	10.8	32	Taylor et al., 2007, Neurobiol Dis., 27:238-47.
43	LRRK2/PARK8	0	NP_940980.2	leucine-rich repeat kinase 2	20.2	56	Liu et al., 2008, Proc Natl Acad Sci U S A. 105:2693-8.
44	HSP70	21	NP_002145.3	heat shock 70kDa protein 4	43.45	172	Shen et al., 2005, J Biol Chem. 280:39962-9.
45	HSP40	17	NP_006136.1	DnaJ (Hsp40) homolog, subfamily B, member 1	59.7	85	Fornai et al., 2004, J Neurochem. 88(1):114-23.
46	RNF11	33	NP_055187.1	ring finger protein 11	41.5	58	Anderson et al., 2007, J Neuropathol Exp Neurol. 66:955-64.
47	PCYT2	1	NP_002852.1	phosphate cytidylyltransferase 2, ethanolamine	35.7	31	Ross et al., 2001, Neuroscience. 102:899-904.

48	PCYT1B	2	NP_004836.2	phosphate cytidylyltransferase 1, choline, beta	61.5	82	Ross et al., 2001, Neuroscience. 102:899-904.
49	PTDSS1	no B	NP_055569.1	phosphatidylserine synthase 1	12.6	60	Ross et al., 2001, Neuroscience. 102:899-904.
