# Supplementary material for: The Role of Intrinsically Unstructured Proteins in Neurodegenerative Diseases
Source: PLoS One. 2009 May 15;4(5):e5566. doi: 10.1371/journal.pone.0005566 (PMC2679209; doi:10.1371/journal.pone.0005566)
Supplement: Table S5 — Alzheimer's disease Protein Dataset. Proteins that contain ≪30 amino acids residues unstructured at a stretch are tabulated here (0.01 MB PDF) [file pone.0005566.s006.pdf]

	Official Symbol	Num	NCBI ID	Official full name	Location	% unstru	Lengt	Reference
1	HADH2/HSD17B1(	5	NP_001032900.1	hydroxysteroid (17-beta) dehydrogenase 10	Xp11.2	0	0	Lustbader et al., 2004, Science. 304:448-52.
2	GAPDH	26	NP_002037.2	glyceraldehyde-3-phosphate dehydrogenase	12p13	0	0	Shalova et al., 2007, Biochim Biophys Acta. 1770:826-32.
3	HMOX1/HSP32	3	NP_002124.1	heme oxygenase (decycling) 1	22q12; 22q13	5.48	13	Kakimura et al., 2002, FASEB J. 16:601-3.
4	COMT	4	NP_000745.1	catechol-O-methyltransferase	22q11.21-q11	1.8	5	Kennedy et al., 2004, J Neural Transm. 111:547-67.
5	AHSG/FETUA	4	NP_001613.2	alpha-2-HS-glycoprotein	3q27	11.1	28	Geroldi et al., 2005, Neurosci Lett. Oct 7;386(3):176-8.
6	NDRG2/SYLD	0	NP_057334.1	NDRG family member 2	14q11.2	18	27	Mitchelmore et al., 2004, Neurobiol Dis., 16:48-58.
7	APOD	6	NP_001638.1	apolipoprotein D	3q26.2-qter	0	0	Carter et al., 2007, Neurochem Int., 50:12-38.
8	ITM2B/FBD	1	NP_068839.1	integral membrane protein 2B	13q14.3	14.2	16	Matsuda et al., 2005, J Biol Chem. 280:28912-6.
9	KLK10/ PRSSL1	0	NP_001070968.1	kallikrein-related peptidase 10	19q13.3-q13.4	7.9	13	Diamandis et al., 2004, Clin Biochem., 7:230-7.
10	BAX	32	NP_004315.1	BCL2-associated X protein	19q13.3-q13.4	3.1	6	Hauptmann et al., 2008, Neurobiol Aging. Feb 21 [Epub a
11	BCL2L2/BCL-W	9	NP_004041.1	BCL2-like 2	14q11.2-q12	0	0	Yao et al., 2005, J Neurosci. 25:1149-58.
12	MSI1	0	NP_002433.1	musashi homolog 1 (Drosophila)	12q24.1-q24.3	12.1	26	Lovell et al., 2005, J Neuropathol Exp Neurol., 64:675-80.
13	PRDX3	3	NP_006784.1	peroxiredoxin 3	10q25-q26	0	0	Kim et al., 2001, J Neural Transm Suppl. 61:223-35.
14	ENO1	3	NP_001419.1	enolase 1, (alpha)	1p36.3-p36.2	4.3	19	Butterfield et al., 2006, Neurobiol Dis., 22:223-32.
15	CDC2/CDK1	82	NP_001777.1	cell division cycle 2, G1 to S and G2 to M	10q21.1	5.3	6	Pie et al., 2002, Acta Neuropathol. 104:369-76.
16	PARK7	7	NP_009193.2	Parkinson disease (autosomal recessive, early onset) 7	1p36.33-p36.1	0	0	Choi et al., 2006, J Biol Chem., 281:10816-24.
17	BACE2/ASP1	0	NP_036237.2	beta-site APP-cleaving enzyme 2	21q22.3	0	0	Stockley et al., 2007, Biochem Soc Trans., 35:574-6.
18	PRDX1	5	NP_002565.1	peroxiredoxin 1	1p34.1	14	28	Kim et al., 2001, J Neural Transm Suppl. 61:223-35.
19	CTSD	7	NP_001900.1	cathepsin D	11p15.5	0	0	Urbanelli et al., 2008, Neurobiol Aging. 29:12-22
20	CDK5	29	NP_004926.1	cyclin-dependent kinase 5	7q36	9.2	27	Wen et al., 2008, J Neurosci. 28:2624-32.
21	TNF	13	NP_000585.2	tumor necrosis factor (TNF superfamily, member 2)	6p21.3	5.1	12	Culpan et al., 2007, Neurobiol Aging. Dec 5 [Epub ahead
22	TTR	16	NP_000362.1	transthyretin (prealbumin, amyloidosis type I)	18q12.1	17	18	Golabek et al., 1995, Neurosci Lett., 191:79-82.
23	HSPB8	14	NP_055180.1	heat shock 22kDa protein 8	12q24.23	31.1	29	Wilhelmus et al., 2006, Acta Neuropathol., 111:139-49.
24	HSPB6	0	NP_653218.1	heat shock protein, alpha-crystallin-related, B6	19q13.12	11.8	12	Wilhelmus et al., 2006, Brain Res., 1089:67-78.
25	APH1A	21	NP_001071096.1	anterior pharynx defective 1 homolog A (C. elegans)	1p36.13-q31.3	0	0	Xie et al., 2005, J Mol Neurosci., 25:67-77.
26	BGN/DSPG1	19	NP_001702.1	biglycan	Xq28	7.06	14	Snow et al., 1995, Arch Biochem Biophys., 320:84-95.

.

ahead of print]

.

of print]
