# Supplementary material for: The Role of Intrinsically Unstructured Proteins in Neurodegenerative Diseases
Source: PLoS One. 2009 May 15;4(5):e5566. doi: 10.1371/journal.pone.0005566 (PMC2679209; doi:10.1371/journal.pone.0005566)
Supplement: Table S6 — Alzheimer's disease Protein Dataset. Proteins that contain ≫30 amino acids residues unstructured at a stretch are tabulated here (0.02 MB PDF) [file pone.0005566.s007.pdf]

	Official Symbol	Numl	NCBI ID	Official full name	Location	% unstructur	Length of	Reference
1	NCSTN/APH2	20	NP_056146.1	nicastrin	1q22-q23	11.09	50	Xie et al., 2005, J Mol Neurosci., 25:67-77.
2	APBB3/SRA	6	NP_006042.3	amyloid beta (A4) precursor protein-binding, family B, memb	5q31	13.78	59	Tanahashi et al., 1999, Neurosci Lett., 261:143-6.
3	APBA3/mint3	2	NP_004877.1	amyloid beta (A4) precursor protein-binding, family A, memb	19p13.3	13.78	93	Tanahashi et al., 1999, Neuroreport., 10:2575-8.
4	PSEN1/AD3/FAD	85	NP_000012.1	presenilin 1 (Alzheimer disease 3)	14q24.3	29.5	71	Finckh et al., 2005, Neurogenetics., 6:85-89.
5	APPBP2/PAT1	23	NP_006371.2	amyloid beta precursor protein (cytoplasmic tail) binding pro	17q21-q23	16.75	62	Kuan et al., 2006, J Biol Chem., 281:40114-23.
6	TGFB1/CED	59	NP_000651.3	transforming growth factor, beta 1	19q13.2; 19q1	17.4	49	Hashimoto et al., 2005, Mol Cell Biol., 25:9304-17.
7	ACHE	6	NP_000656.1	acetylcholinesterase (Yt blood group)	7q22	18.24	50	Silveyra et al., 2008, Mol Cell Biol. Feb 25; [Epub ahead of print]
8	BLMH/ BMH	14	NP_000377.1	bleomycin hydrolase	17q11.2	23	36	Lefterov et al., 2001, Biochem Biophys Res Commun. 283:994-9.
9	FBLN1	16	NP_001987.2	fibulin 1	22q13.31	23.18	53	Ohsawa et al., 2001, J Neurochemistry., 76:1411-1420.
10	NID1	22	NP_002499.2	nidogen 1	1q43	24.53	53	Narindrasorasak et al., 1991, J. Biol. Chem., 266:12878-12883.
11	APPBP1/HPP1	6	NP_001018169.1	amyloid beta precursor protein binding protein 1	16q22	25.84	108	Cao et al., 2001, Science., 293:115-20.
12	SHC1/p66	190	NP_003020.2	SHC (Src homology 2 domain containing) transforming prote	1q21	26.24	55	Xie et al., 2007, J Biol Chem., 282:4318-25.
13	IDE	5	NP_004960.1	insulin-degrading enzyme	10q23-q25	27.5	79	Jean et al., 2007, PLoS ONE., 2:e652.
14	GSN	26	NP_000168.1	gelsolin (amyloidosis, Finnish type)	9q33	29.02	51	Ji et al., 2008, Neuroreport. 19:463-6.
15	HTATIP/TIP60	26	NP_006379.2	HIV-1 Tat interacting protein, 60kDa	11q13	32.55	55	Cao et al., 2001, Science., 293:115-20.
16	NAPB/ SNAP-BETA	0	NP_071363.1	N-ethylmaleimide-sensitive factor attachment protein, beta	20p12.3-p11.2	24.8	33	Yoo et al., 2001, Dement Geriatr Cogn Disord., 12:219-25.
17	F12/HAF	8	NP_000496.1	coagulation factor XII (Hageman factor)	5q33-qter	33.17	94	Yasuhara et al., 1994, Brain Res., 654:234-40.
18	NUMB/S171	29	NP_001005743.1	numb homolog (Drosophila)	14q24.3	34.4	51	Roncarati et al., 2002, Proc Natl Acad Sci U S A., 99:7102-7.
19	COL4A5/ATS/CA54	11	NP_000486.1	collagen, type IV, alpha 5 (Alport syndrome)	Xq22	35.01	128	Kiuchi et al., 2002, Life Sci. 70:1555-64.
20	COL4A1/arresten	16	NP_001836.2	collagen, type IV, alpha 1	13q34	35.17	132	Kiuchi et al., 2002, Life Sci. 70:1555-64.
21	LRP1/APOER	93	NP_002323.2	low density lipoprotein-related protein 1 (alpha-2-macroglobu	12q13-q14	35.21	155	Liu et al., 2007, Neuron., 56:66-78.
22	TGFB2	16	NP_003229.1	transforming growth factor, beta 2	1q41	38.88	77	Harris-White et al., 2004, J Neurosci Res., 77:217-28.
23	COL4A2	16	NP_001837.2	collagen, type IV, alpha 2	13q34	39	183	Kiuchi et al., 2002, Life Sci. 70:1555-64.
24	CAV1/VIP21	78	NP_001744.2	caveolin 1, caveolae protein, 22kDa	7q31.1	40.44	72	Gaudreault et al., 2004, Neurobiol Aging., 25:753-9.
25	ITSN1	24	NP_001001132.1	intersectin 1 (SH3 domain protein)	21q22.1-q22.2	41.13	217	Keating et al., 2006, Ageing Res Rev., 5:388-401.
26	KNG1/BDK	28	NP_000884.1	kininogen 1	3q27	42.39	154	Puchades et al., 2003, Mol. Brain. Res. 118:140-146
27	CASP8	59	NP_001073593.1	caspase 8, apoptosis-related cysteine peptidase	2q33-q34	42.79	70	Rohn et al., 2001, Neurobiol Dis. 8:1006-16.
28	APBB1/FE65	24	NP_001155.1	amyloid beta (A4) precursor protein-binding, family B, memb	11p15	47.18	238	Cao et al., 2001, Science., 293:115-20.
29	COL4A3	16	NP_000082.2	collagen, type IV, alpha 3 (Goodpasture antigen)	2q36-q37	48.26	118	Kiuchi et al., 2002, Life Sci. 70:1555-64.
30	APBB2/FE65L	7	NP_775098.2	amyloid beta (A4) precursor protein-binding, family B, memb	4p14-p13	49.4	201	Cao et al., 2001, Science., 293:115-20.
31	DAB1	22	NP_066566.3	disabled homolog 1 (Drosophila)	1p32-p31	52.43	95	Homayouni et al., 1999, J Neurosci., 19:7507-15.
32	HMOX2	3	NP_002125.3	heme oxygenase (decycling) 2	16p13.3	53.16	48	Takahashi et al., 2000, Neuron. 28:461-73.
33	APBA2/MINT2	12	NP_005494.2	amyloid beta (A4) precursor protein-binding, family A, memb	15q11-q12	55.94	248	Mcloughlin et al., 1999, Eur J Neurosci., 11:1988-94.
34	KLC1/KNS2	10	NP_005543.2	kinesin light chain 1	14q32.3	61.33	105	Kamal et al., 2000, Neuron., 28:449-59.
35	APBA1/MINT1	28	NP_001154.2	amyloid beta (A4) precursor protein-binding, family A, memb	9q13-q21.1	62.84	334	Lee et al., 2003, J Biol Chem. 278:47025-9.
36	CALR	45	NP_004334.1	calreticulin	19p13.3-p13.2	65.46	135	Erickson et al., 2005, Biochem Biophys Res Commun. 332:50-7.
37	COL25A1/CLAC	2	NP_115907.2	collagen, type XXV, alpha 1	4q25	74	137	Kakuyama et al., 2005, Biochemistry. 44:15602-9.
38	SNCA/PARK1	42	NP_000336.1	synuclein, alpha (non A4 component of amyloid precursor)	4q21	31.4	44	Broe et al., 2005, Neurotox Res., 7:69-76.
39	ABCA1	5	NP_005493.2	ATP-binding cassette, sub-family A (ABC1), member 1	9q31.1	18.5	57	Wahrle et al., 2008, J Clin Invest. 118:671-82.
40	ABCA2	0	NP_001597.2	ATP-binding cassette, sub-family A (ABC1), member 2	9q34	16	47	Chen et al., 2004, FASEB J. 18:1129-31.
41	ABL1/p150	121	NP_005148.2	v-abl Abelson murine leukemia viral oncogene homolog 1	9q34.1	41.2	108	Magnani et al., 2007, EMBO J. 26:4546-54.
42	COX10	0	NP_001294.2	COX10 homolog, cytochrome c oxidase assembly protein	17p12-p11.2	20.9	56	Fukui et al., 2007, Proc Natl Acad Sci U S A., 104:14163-8.
43	ATF2/CREB2	48	NP_001871.2	activating transcription factor 2	2q32	41.2	108	Yamada et al., 1997, Brain Res. 749:329-34.

44	EEF2K	0	NP_037434.1	eukaryotic elongation factor-2 kinase	16p12.1	62.4	121	Li et al., 2005, FEBS J., 272:4211-20.
45	UBQLN1	41	NP_038466.2	ubiquilin 1	9q22; 9q21.2-	18.5	49	Thomas et al., 2006, J Biol Chem., 281:26400-7.
46	ADAM10/MADM	2	NP_001101.1	ADAM metallopeptidase domain 10	15q2; 15q22	45.8	81	Asai et al., 2003, Biochem Biophys Res Commun., 301:231-5.
47	RTN4R/NGR	0	NP_075380.1	reticulon 4 receptor	22q11.21	12.8	61	Park et al., 2006, J Neurosci., 26:1386-95.
48	ADRBK1/GRK2	30	NP_001610.1	adrenergic, beta, receptor kinase 1	11q13.1	38.1	98	Takahashi et al., 2006, Neuropathol Exp Neurol., 65:1157-69.
49	PIN1	58	NP_006212.1	protein (peptidylprolyl cis/trans isomerase) NIMA-interacting	19p13	68.7	100	Hamdane et al., 2006, Mol Cell Neurosci., 32:155-60.
50	SNCB	5	NP_001001502.1	synuclein, beta	5q35	32.8	44	Windisch et al., 2002, J Mol Neurosci., 19:63-9.
51	MAPK1/ERK	109	NP_002736.3	mitogen-activated protein kinase 1	22q11.2; 22q1	16.5	65	Nizzari et al., 2007, J Biol Chem., 282:13833-44.
52	COL18A1	9	NP_085059.2	collagen, type XVIII, alpha 1	21q22.3	49.9	121	van Horssen et al., 2002, Brain Pathol., 12:456-62.
53	CXCR3	13	NP_001495.1	chemokine (C-X-C motif) receptor 3	Xq13	17.6	33	Xia et al., 2000, J Neuroimmunol., 108:227-35.
54	GRIA1/GLUH1	26	NP_000818.1	glutamate receptor, ionotropic, AMPA 1	5q33; 5q31.1	22.4	50	Szegedi et al., 2005, Brain Res. 1062:120-6.
55	CTCF	2	NP_006556.1	CCCTC-binding factor (zinc finger protein)	16q21-q22.3	85	168	Wavrant-De Vrièze et al., 1999, Neuroscience Letters., 269: 67-70
56	APOC1	4	NP_001636.1	apolipoprotein C-I	19q13.2	44.5	37	Carter et al., 2007, Neurochem Int., 50:12-38.
57	FASLG/TNFSF6	52	NP_000630.1	Fas ligand (TNF superfamily, member 6)	1q23	35.5	63	Su et al., 2003, Neurobiol Dis., 12:182-93.
58	GRIN2A/NR2A	39	NP_000824.1	glutamate receptor, ionotropic, N-methyl D-aspartate 2A	16p13.2	42.5	288	Sze et al., 2001, J Neurol Sci., 182:151-9.
59	GRIN2B/NR2B	38	NP_000825.1	glutamate receptor, ionotropic, N-methyl D-aspartate 2B	12p12	34.6	188	Sze et al., 2001, J Neurol Sci., 182:151-9.
60	GSK3B	64	NP_002084.2	glycogen synthase kinase 3 beta	3q13.3	17.8	30	Carter et al., 2007, Neurochem Int., 50:12-38.
61	MEOX2	9	NP_005915.2	mesenchyme homeobox 2	7p22.1-p21.3	71.3	119	Wu et al., 2005, Nat Med., 11(9):959-65.
62	MMP2/CLG4	42	NP_004521.1	matrix metallopeptidase 2 (gelatinase A, 72kDa gelatinase,	16q13-q21	71.3	119	Roher et al., 1994, Biochem Biophys Res Commun., 205:1755-61.
63	STMN2	11	NP_008960.2	stathmin-like 2	8q21.13	69.8	125	Okazaki et al., 1995, Neurobiol Aging. 16:883-94.
64	NRG1	14	NP_004486.2	neuregulin 1	8p12	58.9	119	Chaudhury et al., 2003, J Neuropathol Exp Neurol., 62:42-54.
65	MRE11A/ATLD	37	NP_005581.2	MRE11 meiotic recombination 11 homolog A (S. cerevisiae)	11q21	52.8	269	Jacobsen et al., 2004, Brain Res Mol Brain Res., 128:1-7.
66	MAP2K1/MEK1	41	NP_002746.1	mitogen-activated protein kinase kinase 1	15q22.1-q22.3	16.5	65	Zhu et al., 2003, J Neurochem., 86:136-42.
67	KLK7/PRSS6	1	NP_005037.1	kallikrein-related peptidase 7	19q13.33	17.3	44	Diamandis et al., 2004, Clin Biochem., 37:230-7.
68	E2F1	70	NP_005216.1	E2F transcription factor 1	20q11.2	37.9	51	Jordan-Sciutto et al., 2002, J Neuropathol Exp Neurol., 61:358-67.
69	EIF4E2/IF4e	8	NP_004837.1	eukaryotic translation initiation factor 4E family member 2	2q37.1	43.3	58	Li et al., 2005, FEBS J., 272:4211-20.
70	EIF4EBP1/4EBP1	8	NP_004086.1	eukaryotic translation initiation factor 4E binding protein 1	8p12	38.9	46	Li et al., 2005, FEBS J., 272:4211-20.
71	HTR2A/5-HT2A	6	NP_000612.1	5-hydroxytryptamine (serotonin) receptor 2A	13q14-q21	27.3	54	Lai et al., 2005, Psychopharmacology (Berl). 179:673-7.
72	NACA/HSD48	9	NP_005585.1	nascent polypeptide-associated complex alpha subunit	12q23-q24.1	81.8	98	Kim et al., 2002, J Investig Med., 50:293-301.
73	NCAM1/CD56	12	NP_000606.3	neural cell adhesion molecule 1	11q23.1	24	53	Strekalova et al., 2006, Neurobiol Aging., 27(1):1-9.
74	CBS/HIP4	9	NP_000062.1	cystathionine-beta-synthase	21q22.3	20.3	63	Pacheco-Quinto et al., 2006, Neurobiol Dis. 22:651-6.
75	GRIK4/EAA1	1	NP_055434.2	glutamate receptor, ionotropic, kainate 4	11q22.3	11.2	36	Jacob et al., 2007, J Alzheimers Dis., 11:97-116.
76	PADI2/PAD2	0	NP_031391.1	peptidyl arginine deiminase, type II	1p36.13	13.9	50	Ishigami et al., 2005, J Neurosci Res., 80:120-8.
77	GFAP	24	NP_002046.1	glial fibrillary acidic protein	17q21	65.5	107	Ishigami et al., 2005, J Neurosci Res., 80:120-8.
78	SERPINI1/neuroserpin	2	NP_005016.1	serpin peptidase inhibitor, clade I (neuroserpin), member 1	3q26.1	8.7	36	Kinghorn et al., 2006, J Biol Chem., 281:29268-77.
79	GRIK5/EAA2	7	NP_002079.3	glutamate receptor, ionotropic, kainate 5	19q13.2	17.8	50	Jacob et al., 2007, J Alzheimers Dis., 11:97-116.
80	LRP8/APOER2	14	NP_001018064.1	low density lipoprotein receptor-related protein 8, apolipopro	1p34	31.4	95	Fuentealba et al., 2007, Mol Neurodegener. 2:14.
81	SIRT1	6	NP_036370.2	sirtuin (silent mating type information regulation 2 homolog)	10q21.3	52	109	Chen et al., 2005, J Biol Chem. 280:40364-74.
82	CD40/p50	55	NP_001241.1	CD40 molecule, TNF receptor superfamily member 5	20q12-q13.2	36.8	61	Ait-Ghezala et al., 2007, Eur J Neurosci. 25:1685-95.
83	NGFR/CD271	29	NP_002498.1	nerve growth factor receptor (TNFR superfamily, member 16	17q21-q22	37.9	74	Rabizadeh et al., 1994, Proc Natl Acad Sci U S A. 91:10703-6.
84	VSNL1/HLP3	7	NP_003376.2	visinin-like 1	2p24.3	44.5	32	Schnurra et al., 2001, Neurobiol Dis., 8:900-9.
85	CLU/SP-40	26	NP_001822.2	clusterin	8p21-p12	49.6	95	Calero et al., 2005, Subcell Biochem., 38:273-98.
86	FRAP1/MTOR/ RAFT1	17	NP_004949.1	FK506 binding protein 12-rapamycin associated protein 1	1p36.2	14.8	62	Li et al., 2005, FEBS J., 272:4211-20.
87	KCNC4	0	NP_001034663.1	potassium voltage-gated channel, Shaw-related subfamily, r	1p21	34.4	85	Angulo et al., 2004, J Neurochem., 91:547-57
88	FYN/SLK	196	NP_002028.1	FYN oncogene related to SRC, FGR, YES	6q21	36.9	56	Bhaskar et al., 2005, J Biol Chem., 280:35119-25.

89	TUBB3	0	NP_006077.2	tubulin, beta 3	16q24.3	17.1	60	Vijayan et al., 2001, FEBS Lett. 509:375-81.
90	SORL1/LR11	10	NP_003096.1	sortilin-related receptor, L(DLR class) A repeats-containing	11q23.2-q24.2	27.1	182	Offe et al., 2006, J Neurosci. 26:1596-603
91	DHCR24/SELADIN1	0	NP_055577.1	24-dehydrocholesterol reductase	1p33-p31.1	12.7	39	Cecchi et al., 2008, J Cell Mol Med.Jan 11; [Epub ahead of print]
92	BDNF	7	NP_001700.2	brain-derived neurotrophic factor	11p13	47.7	73	Liu et al., 2007, J Huazhong Univ Sci Technolog Med Sci.27:233-6.
93	MME	5	NP_000893.2	membrane metallo-endopeptidase	3q25.1-q25.2	27.7	60	Mohazeri et al., 2002 , J Biol Chem., 277:35460-5.
94	CYP46A1	0	NP_006659.1	cytochrome P450, family 46, subfamily A, polypeptide 1	14q32.1	12.8	47	Carter et al., 2007, Neurochem Int., 50:12-38.
95	PRDX2	0	NP_859428.1	peroxiredoxin 2	19p13.2	18.2	36	Yao et al., 2007, Mol Cell Neurosci. 35:377-82.
96	TFAM	4	NP_003192.1	transcription factor A, mitochondrial	10q21	75.6	186	Hong et al., 2007, Neurochem Res. 32:1483-8.
97	LAMA1	12	NP_005550.2	laminin, alpha 1	18p11.31	17.7	57	Bozzo et al., 2004, Mol Cell Neurosci. 25:1-8.
98	BCHE	1	NP_000046.1	butyrylcholinesterase	3q26.1-q26.2	26	50	Podoly et al., 2008, Neurodegener Dis. 5:232-6.
99	MAPT/TAU	21	NP_005901.2	microtubule-associated protein tau	17q21.1	80	171	Ingram et al., 2002, Trends. Mol. Med., 8:555-62.
100	BACE1/ASP2	4	NP_036236.1	beta-site APP-cleaving enzyme 1	11q23.2-q23.3	8.5	32	Stockley et al., 2007, Biochem Soc Trans., 35:574-6.
101	PSEN2	46	NP_000438.2	presenilin 2 (Alzheimer disease 4)	1q31-q42	32.7	66	Finckh et al., 2005, Neurogenetics., 6:85-89.
102	IFNG	5	NP_000610.2	interferon, gamma	12q14	43.3	46	Yamamoto et al., 2007, Am J Pathol. 170:680-92.
103	BAD/BCL2L8	52	NP_004313.1	BCL2-antagonist of cell death	11q13.1	87.6	185	Kitamura et al., 1998, Brain Res. 780:260-9.
104	IL1B	5	NP_000567.1	interleukin 1, beta	2q14	38.2	36	Tachida et al., 2008, J Neurochem. 104:1387-93.
105	BPTF	3	NP_004450.3	bromodomain PHD finger transcription factor	17q24.3	67.4	367	Jordan-Sciutto et al., 2001, Mech Ageing Dev. 123:11-20.
106	ACE	1	NP_000780.1	angiotensin I converting enzyme (peptidyl-dipeptidase A) 1	17q23.3	20	123	Zou et al., 2007, J Neurosci. 27:8628-35.
107	NOS3	12	NP_000594.2	nitric oxide synthase 3 (endothelial cell)	7q36	26.1	41	Gentile et al., 2004, J Biol Chem. 279:48135-42.
108	VEGFA	31	NP_001020537.2	vascular endothelial growth factor A	6p12	59.9	81	Mateo et al., 2007, Acta Neurol Scand. 116(1):56-8.
109	APOE/AD2	20	NP_000032.1	apolipoprotein E	19q13.2	54.5	57	Lambert et al., 2007, Psychoneuroendocrinology., 32:S62-70.
110	APP/AD1	101	NP_000475.1	amyloid beta (A4) precursor protein (peptidase nexin-II, Alzh	21q21.2; 21q2	55	112	Taylor et al., 2002, Science. 296, 1991-1995.
111	IDE	5	NP_004960.2	insulin-degrading enzyme	10q23-q25	26.5	79	Venugopal et al., 2007, Neurochem Res. 32:2225-34.
112	HSPB2	5	NP_001532.1	heat shock 27kDa protein 2	11q22-q23	19.7	36	Wilhelmus et al., 2006, Brain Res., 1089:67-78.
113	CRYAB	11	NP_001876.1	crystallin, alpha B	11q22.3-q23.1	29.1	33	Smith et al., 2005, Int J Hyperthermia. 21:421-31.
114	GRB2	410	NP_002077.1	growth factor receptor-bound protein 2	17q24-q25	64	73	Nizzari et al., 2007, J Biol Chem. 282:13833-44.
