# Supplementary material for: The Role of Intrinsically Unstructured Proteins in Neurodegenerative Diseases
Source: PLoS One. 2009 May 15;4(5):e5566. doi: 10.1371/journal.pone.0005566 (PMC2679209; doi:10.1371/journal.pone.0005566)
Supplement: Table S7 — Control dataset 2 consisting of metabolic enzymes with known structures (0.01 MB PDF) [file pone.0005566.s008.pdf]

	Protein	Length	Fold-Index	Total	Maximum
1	P07327.seq	374	0.2842642	19	19
2	Q13131.seq	374	0.2842642	0	0
3	Q99714.seq	261	0.3097617	0	0
4	Q8WYK0.sec	555	0.1867897	98	47
5	P00374.seq	187	0.097867	73	38
6	mgat3.seq	531	0.0872857	182	90
7	P04062.seq	536	0.1985996	8	8
8	P09467.seq	337	0.2068638	25	19
9	alg5.seq	324	0.1825572	47	10
10	Q9BXS1.seq	227	0.0692418	33	27
11	P35557.seq	465	0.0921502	102	52
12	P00492.seq	217	0.1872293	0	0
13	Q14376.seq	348	0.1694869	0	0
14	1vis.seq	396	0.244944	27	6
15	P52788.seq	366	0.1184862	62	18
16	Q15046.seq	596	0.0823052	207	99
17	Q9HA47.seq	277	0.0917689	70	37
18	P00387.seq	300	0.1837317	52	25
19	O15067.seq	300	0.1837317	0	0
20	Q96C23.seq	342	0.1272651	10	10
21	O14556.seq	408	0.1692286	54	29
22	man2a1.seq	1144	0.1372926	275	56
23	Q9NR19.seq	701	0.1386588	173	72
24	P00966.seq	412	0.1229385	66	22
25	Q07131.seq	469	0.1014412	57	18
26	P19367.seq	917	0.1699665	145	34
27	P17707.seq	334	0.1248288	28	28
28	P21964.seq	271	0.2540431	5	5
29	P51659.seq	421	0.1268755	71	54
30	Q96EK6.seq	184	0.1811891	0	0
31	Q16831.seq	310	0.2424101	0	0
32	P20839.seq	514	0.2313364	21	21
33	P21695.seq	348	0.252544	9	9
34	P30838.seq	453	0.1700108	105	44
35	Q8TET4.seq	914	0.0998584	262	85
36	Q8TDX5.seq	336	0.1746738	49	49
37	P60891.seq	317	0.2436855	0	0
38	P00491.seq	289	0.1830792	36	27
39	P04035.seq	888	0.2615861	46	30
40	P23921.seq	792	0.1273887	129	66
41	P16118.seq	471	0.082822	124	56
42	P49789.seq	146	0.0993537	62	56
43	Q00796.seq	356	0.2439455	0	0
44	P46952.seq	286	0.1005294	55	28
45	P10746.seq	265	0.1827754	8	8
46	P26639.seq	723	0.0512763	295	80
47	P30566.seq	484	0.1639007	35	22
48	P29803.seq	388	0.132762	94	59
49	Q16719.seq	465	0.1867806	60	34

50	O43612.seq	131	0.1957935	14	8
51	P52209.seq	482	0.1916025	32	17
52	P11310.seq	421	0.1268755	71	54
53	P36959.seq	345	0.2121192	25	25
54	Q9P1U0.seq	126	0.0912272	44	44
55	Q12882.seq	1025	0.2184892	140	70
56	Q96RQ3.seq	725	0.1407577	118	32
57	O43451.seq	725	0.1407577	0	0
58	Q13907.seq	284	0.1307243	28	12
59	P78330.seq	225	0.1800848	0	0
60	Q14117.seq	519	0.1764773	93	56
61	P40126.seq	519	0.1376521	119	65
62	P46597.seq	345	0.1813664	35	23
63	P30837.seq	517	0.1659118	34	34
64	P51857.seq	326	0.1263599	39	32
65	Q9BWD1.sec	397	0.2991394	14	9
66	Q9HAW8.sec	507	0.1415676	141	43
67	P11172.seq	480	0.2632081	0	0
68	Q96Q40.seq	384	0.1587067	46	46
69	P14174.seq	114	0.2270286	0	0
70	Q13956.seq	83	0.0010438	49	49
71	P19801.seq	751	0.1103197	218	63
72	P04183.seq	234	0.2032614	41	14
73	O43708.seq	216	0.1887376	0	0
74	mgat1.seq	445	0.1237803	74	33
75	Q8NBZ7.seq	412	0.2408508	0	0
76	O95394.seq	542	0.1303366	68	22
77	P10515.seq	614	0.2058567	25	8
78	Q6ZMQ8.seq	1374	0.0496577	483	117
79	P07741.seq	179	0.2593702	0	0
80	P42126.seq	302	0.2392198	33	33
81	Q01581.seq	520	0.1275603	91	43
82	P27708.seq	2225	0.2008736	167	74
83	O75874.seq	414	0.1154395	107	38
84	P00813.seq	362	0.0898424	113	53
85	Q9UGM6.sec	360	0.1588825	61	31
86	Q16881.seq	499	0.1818813	76	43
87	Q9UIJ7.seq	226	0.1117971	75	39
88	P49961.seq	510	0.1980455	10	5
89	P22830.seq	423	0.1134131	106	31
90	P19961.seq	650	0.1339914	162	57
91	P50583.seq	292	0.0900526	108	40
92	P19623.seq	302	0.1490934	0	0
93	Q9H227.seq	735	0.1846942	38	13
94	P22234.seq	424	0.1775711	49	44
95	P49327.seq	2511	0.2047934	145	51
96	P00367.seq	558	0.1489868	59	22
97	alg12.seq	488	0.3615739	0	0
98	Q9BYC2.seq	517	0.2253262	50	34
99	P49915.seq	693	0.1843648	51	23

100	P53602.seq	400	0.2087493	44	32
101	st6gal1.seq	406	0.074119	128	59
102	Q9UL12.seq	319	0.2962091	0	0
103	P22680.seq	504	0.1667353	35	20
104	Q14353.seq	236	0.1640168	7	7
105	Q969G6.seq	162	0.1190053	28	28
106	P08236.seq	763	0.191206	25	19
107	P11086.seq	282	0.1606725	69	34
108	P11413.seq	514	0.1180077	56	18
109	P21397.seq	527	0.1515142	54	25
110	Q9UJ70.seq	343	0.2185699	0	0
111	P31327.seq	1994	0.1892543	79	23
112	Q9Y315.seq	318	0.1964253	26	26
113	Q02318.seq	531	0.1673204	75	55
114	Q8WVQ1.sec	401	0.1140324	88	36
115	Q13232.seq	169	0.2429095	0	0
116	P53350.seq	603	0.1168101	106	39
117	P47989.seq	1332	0.1797693	106	30
118	Q15274.seq	297	0.3281282	0	0
119	P15121.seq	315	0.1547934	0	0
120	p19971.seq	482	0.2474161	34	34
121	P16278.seq	677	0.1696972	119	39
122	P31939.seq	592	0.1995508	112	32
123	Q16775.seq	260	0.1210202	43	37
124	P11498.seq	1178	0.1789604	59	29
125	Q9Y6T7.seq	804	0.1056535	298	49
126	P37837.seq	337	0.1580671	41	29
127	Q8N909.seq	420	0.1127959	90	51
128	gcs1.seq	836	0.1499394	113	30
129	P49419.seq	511	0.2190056	46	32
130	P35520.seq	550	0.1432153	111	62
131	P41250.seq	739	0.1472871	75	36
132	Q06210.seq	698	0.1487449	154	51
133	O43252.seq	624	0.1039929	184	54
134	P13807.seq	530	0.2595014	0	0
135	P04040.seq	526	0.0550298	154	82
136	P32929.seq	405	0.198845	32	27
137	fut8.seq	446	0.0927777	119	43
138	P06280.seq	429	0.1409351	82	41
139	P28330.seq	430	0.171123	77	33
140	Q16222.seq	522	0.1271598	62	24
141	P23743.seq	732	0.1504205	47	13
142	O60547.seq	372	0.1211126	58	28
143	P19835|.seq	742	0.1339585	246	73
144	P09622.seq	509	0.2306091	0	0
145	b4galt3.seq	393	0.063321	145	60
146	Q02218.seq	1002	0.1281192	166	51
147	P30793.seq	250	0.0802218	83	78
148	P04181.seq	439	0.2091062	12	12
149	Q15126.seq	192	0.0924285	75	45

150	Q9NP81.seq	518	0.1117448	123	61
151	O95050.seq	263	0.1576302	71	32
152	P15291.seq	397	0.1630942	57	49
153	ddost.seq	456	0.213617	18	10
154	Q05524.seq	458	0.1642217	53	29
155	O60701.seq	494	0.1700114	29	8
156	P60174.seq	248	0.198425	0	0
157	P20711.seq	960	0.224954	158	39
158	P08397.seq	361	0.1726092	64	27
159	ganab.seq	944	0.1060401	233	77
160	Q16853.seq	763	0.182348	61	53
161	P14920.seq	347	0.131874	68	32
162	mgat4b.seq	548	0.1381391	167	57
163	P17516.seq	323	0.1436554	32	32
164	P04818.seq	312	0.1053578	125	47
165	P35914.seq	650	0.2839837	0	0
166	P33121.seq	698	0.2082393	40	15
167	1gtk.seq	260	0.1125913	58	34
168	Q16854.seq	277	0.1418503	0	0
169	P14618.seq	530	0.1969193	7	7
170	P12277.seq	381	0.0718519	70	64
171	Q02127.seq	395	0.1531241	30	18
172	P40925.seq	333	0.226267	8	8
173	p1151.seq	502	0.2392792	5	5
174	P55263.seq	362	0.1320605	37	15
175	75600.seq	419	0.2305009	0	0
176	man1a1.seq	653	0.1409995	143	69
177	P28340.seq	1107	0.1588334	145	80
178	mgat4a.seq	535	0.1643218	113	41
179	Q8N5Z0.seq	425	0.195797	38	38
180	P48728.seq	403	0.2110788	24	18
181	O60701.seq	494	0.1700114	29	8
182	P08237.seq	779	0.1824267	90	34
183	P11216|phos	842	0.1296599	219	57
184	alg10.seq	317	0.3569768	0	0
185	P50440.seq	423	0.1033133	103	24
186	P15289.seq	507	0.2174829	12	7
187	Q03154.seq	408	0.1242035	30	18
188	Q9BXI3.seq	368	0.0961439	113	55
189	Q9BY32.seq	194	0.1734039	51	35
190	P51570.seq	392	0.205456	35	17
191	P35790.seq	457	0.0929159	132	60
192	mgat2.seq	447	0.1183804	71	29
193	Q9UDI9.seq	63	0.1074178	27	27
194	P13716.seq	330	0.2214379	0	0
195	Q9H477.seq	322	0.2566056	0	0
196	alg6.seq	507	0.4208897	18	18
197	Q16613.seq	207	0.2392437	5	5
198	Q8N142.seq	457	0.150668	77	26
199	P36871.seq	561	0.1944017	104	44

200	Q93099.seq	445	0.1244718	72	44
201	P54687.seq	386	0.1572624	13	13
202	P17735.seq	454	0.2065421	68	62
203	P51606.seq	417	0.1086809	62	37
204	Q96EY8.seq	250	0.1138141	46	46
205	P21549.seq	392	0.2119551	38	18
206	Q14749.seq	294	0.1560788	24	16
207	Q9BUT1.seq	245	0.2341412	13	13
208	b4galt1.seq	398	0.1626691	57	49
209	P17752.seq	444	0.0928853	128	60
210	P17812.seq	591	0.1297595	120	69
211	Q04760.seq	183	0.0594549	46	29
212	Q8NFW8.seq	434	0.1088929	120	61
213	P27707.seq	260	0.0353386	96	53
214	P06132.seq	367	0.1797598	47	41
215	Q92947.seq	438	0.1731855	73	28
216	P05091.seq	517	0.1973173	42	27
217	b4galt2.seq	372	0.118567	114	88
218	alg8.seq	467	0.3887442	0	0
219	Q9BYV1.seq	514	0.2142899	0	0
220	P31040.seq	664	0.1563467	107	47
221	P00480.seq	354	0.1543195	21	9
222	P35573.seq	660	0.0818368	232	87
223	P18669.seq	253	0.0806231	99	60
224	P13489.seq	461	0.211627	0	0
225	Q9UBQ7.seq	328	0.2368742	0	0
226	P22413.seq	660	0.15592	67	59
227	P04075.seq	363	0.1502947	7	7
228	P32754.seq	392	0.0930219	37	13
229	P00505.seq	430	0.1512494	14	14
230	P20132.seq	328	0.2936174	0	0
231	P35558.seq	622	0.1396378	171	36
232	Q9Y2T3.seq	454	0.1574533	38	19
233	mgat5.seq	741	0.1453579	143	47
234	fut11.seq	492	0.0777716	191	78
235	P06744.seq	557	0.128277	116	41
236	P11926.seq	461	0.152313	67	32
237	Q6ZMR3.seq	332	0.2320307	5	5
238	P07311.seq	98	0.0309142	64	37
239	P50053.seq	298	0.1951332	30	30
240	Q93088.seq	406	0.119943	94	83
241	Q9Y617.seq	370	0.2448272	6	6
242	O15382.seq	392	0.2038535	33	16
243	Q9Y223.seq	722	0.2348933	8	8
244	P49961|.seq	510	0.1980455	10	5
245	alg1.seq	464	0.252656	37	15
246	P26640.seq	1264	0.1573732	240	97
247	Q16774|.seq	196	0.1078337	70	28
248	P00568.seq	194	0.0769079	47	22
249	P26440.seq	423	0.202422	25	25

250	P23919.seq	212	0.1377715	0	0
251	P00558.seq	416	0.20871	0	0
252	Q04446.seq	511	0.1085463	138	29
253	P80404.seq	500	0.1534322	60	18
254	O75061.seq	913	0.0658635	324	115
255	Q16760.seq	1214	0.1060799	451	93
256	Q99798.seq	780	0.1377595	180	59
257	P30085.seq	196	0.0490622	91	56
258	2v4w.seq	508	0.1354317	96	45
259	P33316.seq	252	0.0818142	114	69
260	P04424.seq	464	0.1679644	50	50
261	P07954.seq	510	0.2088826	37	27
262	P50416|.seq	773	0.1464449	145	45
263	P14550.seq	324	0.1466656	46	26
264	Q08828.seq	1119	0.2761597	116	30
