# Supplementary material for: The Role of Intrinsically Unstructured Proteins in Neurodegenerative Diseases
Source: PLoS One. 2009 May 15;4(5):e5566. doi: 10.1371/journal.pone.0005566 (PMC2679209; doi:10.1371/journal.pone.0005566)
Supplement: Table S8 — Control dataset 3 consisting of proteins involved in Breast Cancer (0.08 MB PDF) [file pone.0005566.s009.pdf]

	Protein	Length	Fold-Index	Total	Maximum
1	Q6IAI2_HUMAN.seq	246	-0.1540303	177	87
2	Q6P5Z4_HUMAN.seq	870	0.0643328	255	110
3	ERGI3_HUMAN.seq	383	0.1343844	108	102
4	Q8IU77_HUMAN.seq	35	-0.0676297	35	35
5	Q5XLT4_HUMAN.seq	1841	-0.038691	1291	274
6	Q6TV06_HUMAN.seq	802	-0.0051102	459	109
7	Q05GC8_HUMAN.seq	1220	0.0027162	627	189
8	Q8IU64_HUMAN.seq	22	-0.0833448	22	22
9	Q8IU82_HUMAN.seq	38	0.1212589	0	0
10	BRCA2_HUMAN.seq	3418	0.0331937	1649	108
11	Q96CD4_HUMAN.seq	466	0.101435	102	102
12	B4DGB5_HUMAN.seq	868	0.0635214	250	110
13	Q3YB50_HUMAN.seq	35	0.0098437	0	0
14	A4D1X9_HUMAN.seq	695	-0.0326751	440	128
15	ABCG2_HUMAN.seq	655	0.2848948	66	21
16	NFIP1_HUMAN.seq	221	0.2179977	74	74
17	LHR2A_HUMAN.seq	786	0.1825668	70	17
18	Q5TBJ7_HUMAN.seq	3418	0.0333839	1647	108
19	B3KWD7_HUMAN.seq	888	0.0672029	246	100
20	PKHA8_HUMAN.seq	519	0.105636	114	80
21	SEPT1_HUMAN.seq	367	0.0466532	151	60
22	GREB1_HUMAN.seq	1949	0.1554397	429	130
23	RHBT2_HUMAN.seq	727	0.1333037	144	88
24	Q64FK1_HUMAN.seq	15	-0.0080173	15	15
25	K0100_HUMAN.seq	2235	0.1546555	572	62
26	CQ071_HUMAN.seq	991	0.0935627	362	107
27	PP14C_HUMAN.seq	165	-0.0114089	127	127
28	PRP31_HUMAN.seq	499	0.0745913	200	82
29	PBOV1_HUMAN.seq	135	0.136925	29	18
30	Q8IZK4_HUMAN.seq	15	-0.8455782	15	15
31	Q5TEW3_HUMAN.seq	734	0.0877027	230	64
32	ANR17_HUMAN.seq	2603	0.106311	761	115
33	Q9H283_HUMAN.seq	43	-0.1921979	43	43
34	BCAR3_HUMAN.seq	825	0.0661295	330	116
35	B4DEV4_HUMAN.seq	916	0.0681863	272	110
36	B4DIW5_HUMAN.seq	888	0.0575741	262	110
37	NRG1_HUMAN.seq	640	-0.0184784	377	119
38	B3KW85_HUMAN.seq	841	0.0653485	250	110
39	Q9NQR3_HUMAN.seq	28	-0.4333386	28	28
40	K1967_HUMAN.seq	923	0.0404789	369	122
41	AN30B_HUMAN.seq	1477	-0.0376387	1106	168
42	Q9H287_HUMAN.seq	132	-0.4034132	132	132
43	Q3LRH8_HUMAN.seq	1822	-0.0434929	1307	274
44	Q3YB51_HUMAN.seq	37	-0.028921	37	37
45	B3KWE2_HUMAN.seq	660	0.0756339	155	110
46	SFXN4_HUMAN.seq	337	0.2485168	0	0
47	B3KNL6_HUMAN.seq	605	0.0968658	198	64
48	Q64FK2_HUMAN.seq	13	0.1177175	0	0
49	A4D120_HUMAN.seq	166	0.1499059	16	8
50	HEAT6_HUMAN.seq	1181	0.2180099	84	68
51	Q05GC6_HUMAN.seq	1200	0.0001131	622	189
52	B3KWS6_HUMAN.seq	323	0.0804871	110	110

53	A8K5Y2_HUMAN.seq	695	-0.0315619	432	120
54	BCAS1_HUMAN.seq	584	-0.0834135	441	149
55	Q9H279_HUMAN.seq	188	0.0973556	64	53
56	BRMS1_HUMAN.seq	246	-0.1540303	177	87
57	NCOA3_HUMAN.seq	1424	0.0060551	831	97
58	Q64FK3_HUMAN.seq	91	-0.1726907	91	91
59	Q3YB52_HUMAN.seq	33	0.1414988	0	0
60	B4DLQ5_HUMAN.seq	275	-0.0458013	148	52
61	B2RBL9_HUMAN.seq	870	0.0656374	231	110
62	PSMD6_HUMAN.seq	389	0.1083484	98	60
63	BRCA1_HUMAN.seq	1863	-0.0389695	1307	274
64	B3KU43_HUMAN.seq	265	-0.0190485	172	52
65	STRAD_HUMAN.seq	431	0.1447216	90	36
66	Q4EW25_HUMAN.seq	166	-0.1345516	164	128
67	Q7Z606_HUMAN.seq	1141	-0.0761719	919	143
68	Q8WZ76_HUMAN.seq	103	0.2437055	0	0
69	Q9H4L2_HUMAN.seq	54	-0.0858337	54	54
70	Q8IZK2_HUMAN.seq	28	-0.4731243	28	28
71	SYUG_HUMAN.seq	127	0.030294	34	34
72	Q3YB53_HUMAN.seq	39	-0.1036114	39	39
73	Q6TV07_HUMAN.seq	1780	-0.0200546	1130	255
74	AKP13_HUMAN.seq	2813	0.0017787	1476	147
75	KIF15_HUMAN.seq	1388	-0.031133	879	291
76	Q3YB49_HUMAN.seq	39	-0.0250602	39	39
77	BCAR1_HUMAN.seq	870	0.0652932	250	110
78	AN30A_HUMAN.seq	1397	-0.0300709	953	246
79	CALL4_HUMAN.seq	196	0.0460442	99	64
80	BRM1L_HUMAN.seq	323	-0.1274714	230	93
81	Q9BX99_HUMAN.seq	715	0.0550041	278	84
82	A1YBP1_HUMAN.seq	2649	0.0179225	1324	108
83	TFF1_HUMAN.seq	84	0.1855448	28	28
84	Q3LRJ0_HUMAN.seq	1863	-0.0385988	1307	274
85	Q6FHG5_HUMAN.seq	127	0.030294	34	34
86	ZNHI6_HUMAN.seq	470	-0.0281145	342	189
87	B4DWK2_HUMAN.seq	501	0.1047691	125	56
88	Q9H4L3_HUMAN.seq	56	0.1917977	0	0
89	Q9H281_HUMAN.seq	352	0.0729461	118	64
90	SVEP1_HUMAN.seq	3574	0.1136757	665	58
91	Q7KYU6_HUMAN.seq	233	0.1413277	44	44
92	MAGD2_HUMAN.seq	606	0.0512514	220	164
93	NCOA6_HUMAN.seq	2063	0.0140916	1091	164
94	Q6QEF7_HUMAN.seq	888	0.0751234	236	101
95	SGOL1_HUMAN.seq	561	-0.0844558	416	261
96	BLID_HUMAN.seq	108	0.2351899	5	5
97	Q9H273_HUMAN.seq	205	0.0040767	95	51
98	Q9H282_HUMAN.seq	40	0.0971303	0	0
99	BIN2_HUMAN.seq	565	-0.0620719	456	119
100	BCA3_HUMAN.seq	210	0.0470495	104	55
101	Q13559_HUMAN.seq	34	0.2897296	0	0
102	Q8IZK3_HUMAN.seq	28	-0.4447339	28	28
103	CF204_HUMAN.seq	805	-0.0398486	605	306
104	ARI4B_HUMAN.seq	1312	-0.1440695	1040	541
105	B3KP06_HUMAN.seq	825	0.0699673	330	116

106	O95958_HUMAN.seq	331	0.0533869	124	63
107	A1A518_HUMAN.seq	179	0.304999	0	0
108	A4D1X8_HUMAN.seq	653	-0.0274279	408	128
109	Q8IU58_HUMAN.seq	21	-0.2018851	21	21
110	Q3LRJ6_HUMAN.seq	1863	-0.0389695	1307	274
111	LATH_HUMAN.seq	179	0.304999	0	0
112	CF125_HUMAN.seq	126	-0.0826806	126	126
113	Q8IZT7_HUMAN.seq	28	-0.4465972	28	28
114	TREF1_HUMAN.seq	1200	0.0001131	622	189
115	AGR3_HUMAN.seq	166	0.1499059	16	8
116	B4DES0_HUMAN.seq	712	-0.0180311	405	285
117	FA84B_HUMAN.seq	310	-0.02440368[illegible]	216	94
